# Identification of shared viral sequences in peat moss metagenomes reveals elements of a possible *Sphagnum* core virome

**DOI:** 10.1186/s40793-025-00719-0

**Published:** 2025-06-05

**Authors:** Elizabeth R. Denison, Helena L. Pound, Eric R. Gann, Naomi E. Gilbert, David J. Weston, Dale A. Pelletier, Steven W. Wilhelm

**Affiliations:** 1 Department of Microbiology, University of Tennessee, Knoxville, TN USA; 2 Physical and Life Sciences Directorate, Lawrence Livermore National Laboratory, Livermore, CA USA; 3 Biosciences Division, Oak Ridge National Laboratory, Oak Ridge, TN USA

**Keywords:** Plant Microbiome, Peatland, Metagenomics, Metatranscriptomics, Viral Ecology

## Abstract

**Background:**

Viruses are an understudied component of plant microbiomes. Identifying viruses that are shared between individual plants, or members of the “core virome”, could reveal stable viral populations with the potential to modulate the composition and function of the microbiome. Here, we examined the virome associated with *Sphagnum* mosses, a keystone species that has direct influence over the fate of peatland carbon stores. We analyzed bulk metagenomes and metatranscriptomes generated from *Sphagnum* field samples collected over a ten-month period to identify virus-like sequences shared among plants.

**Results:**

Individual *Sphagnum* samples harbored distinct DNA and RNA viromes where only a small percentage (< 1%) of the total number of identified viral contigs were shared among all samples. Based on taxonomic classification, the shared viral contigs represent bacterial viruses, or phage (*Caudoviricetes*), as well as viruses of eukaryotes, namely nucleocytoplasmic large DNA viruses (*Nucleocytoviricota*) and RNA viruses (*Riboviria*). We linked the shared phage-like contigs to viral regions within sequenced genomes of bacterial taxa that are members of the *Sphagnum* core microbiome, suggesting that these contigs represent temperate phage or degraded prophage. The putative nucleocytoplasmic large DNA viruses and RNA viruses were phylogenetically diverse and showed sequence similarity to viruses associated with a broad range of hosts and environmental sources.

**Conclusions:**

The identification of shared viral contigs suggested that, despite the compositional heterogeneity between samples, *Sphagnum* mosses may harbor a core virome. Future work validating the presence of the core virome is warranted as it may aid in understanding how persistent viruses impact microbiome ecology and symbiont evolution within this climatically relevant keystone species.

**Supplementary Information:**

The online version contains supplementary material available at 10.1186/s40793-025-00719-0.

## Background

Peatlands serve as one of Earth’s largest carbon sinks by sequestering about one-third of all terrestrial carbon in the form of peat, or partially decayed organic matter [1]. The majority of global peatland carbon stocks are stored in northern peatlands, where low mean temperatures, nutrient limitation, and anoxic conditions slow the rate of decomposition and promote the accumulation of organic matter [2]. However, climate warming threatens to reduce carbon capture, mobilize existing carbon stores, and ultimately transition peatlands from carbon sinks to sources of atmospheric carbon [3–5]. Carbon sequestration in northern peatlands is largely engineered by *Sphagnum* mosses, or peat mosses, which are a dominant feature of peatland vegetation [6]. *Sphagnum* spp. both thrive in and promote the nutrient-poor and acidic conditions that contribute to slow decay rates in peatlands [7, 8]. Recalcitrant *Sphagnum* litter and *Sphagnum*-derived antimicrobials are also thought to promote peat accumulation [9, 10], further supporting its role in ecosystem stability and global carbon sequestration.

The productivity and ecological dominance of *Sphagnum* is tied to its associated microbial community [11, 12]. *Sphagnum* spp. harbor diverse communities of bacteria and microbial eukaryotes (protists and fungi) [11, 13], where certain functional guilds perform roles that are important for moss health. For example, clades of diazotrophic bacteria supply fixed nitrogen to the moss in return for carbohydrates and sulfur-rich metabolites [14, 15]. Far less is known about the eukaryotic communities associated with *Sphagnum*, but they are thought to influence the moss microbiome *via* grazing and nutrient transformation [16, 17]. *Sphagnum* symbionts are involved in a range of other roles including pathogen suppression [18], stress tolerance [12, 19], and disease [20]. Given that the microbiome influences moss productivity and the peatland carbon balance, it is important to understand factors that influence the composition and function of the *Sphagnum* microbiome.

Viral infection shapes microbial community structure and function through lysis [21], lateral gene transfer [22], and by altering the physiology of infected cells [23]. Viruses are also recognized mediators of carbon and nutrient cycles [24], yet they are an often-overlooked component of land plant microbiomes. Research on plant viromes has historically focused on viruses that infect the host plant [25], but recent work has begun to uncover the roles of viruses within plant microbiomes. Plant-associated viruses can influence bacterial diversity, suppress plant pathogens, and have the potential to modulate plant-microbe interactions [26–29].

Identifying “core” guilds within plant microbiomes can reveal important microbial functions that impact plant health as well as plant-microbe evolutionary relationships [30]. For example, in *Sphagnum*, defining core bacteria has further highlighted the beneficial relationship between peat moss and diazotrophic clades [31, 32]. Metatranscriptomic analysis has suggested that viruses actively infect bacteria and microbial eukaryotes in the moss microbiome [33], but no studies have directly examined if there are viral communities shared between different *Sphagnum* samples. Expanding our definition of the *Sphagnum* core microbiome to include viruses may aid in understanding the ecology and evolution of the peat moss microbiome.

Here, we leveraged paired bulk metagenomes and metatranscriptomes from living *Sphagnum* tissue collected over a ten-month period (August 2016 to June 2017) to assess the distribution of viral sequences across wild plants. Metagenomic assembly allowed for the identification of DNA virus contigs and viral metagenome-assembled genomes (MAGs), while the metatranscriptomes were used to search for RNA virus genomes. Viral contig distribution was generally patchy across the samples, but a small percentage of the identified contigs were detected in all samples and may constitute part of a *Sphagnum* core virome. The shared phage-like contigs (*n* = 5) were linked to viral regions within sequenced genomes of the *Acetobacteraceae* and *Acidobacteriaceae*, two families that contain taxa previously found to be members of the *Sphagnum* core microbiome. These phage-like contigs may therefore represent temperate phage or degraded prophage-derived regions in bacterial genomes within the core microbiome. Additionally, phylogenetic analysis indicated that the shared RNA virus contigs (*n* = 9) belonged to six different virus families, and that common NCLDV MAGs (*n* = 2) were related to the *Pimascovirales* and *Asfuvirales* orders. Lastly, our findings suggested that some of the possible members of the core virome may be maintained in the microbiome through strictly intracellular replication strategies based on their taxonomic assignment and lack of recognizable capsid proteins.

## Materials and methods

### Site description and sample collection

*Sphagnum* samples were collected from the Spruce and Peatland Responses Under Changing Environments (SPRUCE) field site located at the Marcell Experimental Forest S1 bog in northern Minnesota, USA (https://mnspruce.ornl.gov). The S1 bog is a nutrient-deficient and acidic (pH 3.5–4) *Sphagnum*-dominated bog [34]. Eight individual plants were collected at four different dates over a ten-month period: 1 August 2016 (*n* = 1), 10 November 2016 (*n* = 3), 18 April 2017 (*n* = 2), and 26 June 2017 (*n* = 2) (Table S1). Samples were collected from hollow (*n* = 6) and lawn (*n* = 2) micro-topographies (Table S1). Only living *Sphagnum* tissue was collected (*i*.*e*., capitula and approximately 3 cm of living stem). Tissue samples were flash frozen in dry ice-alcohol baths and shipped to Oak Ridge National Laboratory on dry ice where they were stored at -80° C until further processing.

### Nucleic acid extraction, sequence processing, and contig assembly

Genomic DNA extractions were performed using DNeasy Plant Pro Kit (QIAGEN) according to the manufacturer’s instructions. Total RNA was extracted using a method combining CTAB lysis buffer and the Spectrum Total Plant RNA Kit (Sigma) as described previously [35]. Library construction and sequencing were performed by Hudson Alpha (Huntsville, AL, USA). Eight PCR-free metagenome libraries (one per tissue sample) were constructed using the TruSeq DNA PCR-Free LT Library Prep Kit (Illumina). Sixteen metatranscriptome libraries (two replicates per tissue sample) were prepared using the ScriptSeq RNA-Seq Library Preparation Kit and were depleted with Ribo-Zero yeast, Ribo-Zero bacteria and Ribo-Zero plant (Illumina). Libraries were quantified by Qubit (Invitrogen) for concentration, by Agilent Bioanalyzer (Agilent Technologies) for fragment size, and by qPCR for determining the optimal loading concentration. Libraries were sequenced on the Illumina HiSeq 2500 platform (2 × 250 bp reads). DNA libraries were pooled two per channel and RNA libraries were pooled four per channel.

Raw reads were trimmed to remove adapters and quality scores < 0.3 using CLC Genomics Workbench v12.0 (QIAGEN). To reduce the number of host plant reads prior to assembly, metagenome and metatranscriptome reads that mapped to the *S. fallax* v1.1 (GCA_021442195.1) or *S. magellanicum* v1.1 (GCA_021904315.1) genome were removed from the libraries. Read mapping was performed using BBMap v38.90 with the default settings [36]. Unmapped reads comprised 64.0% ± 4.8% (mean ± standard error) of reads in the metagenome libraries and 28.5% ± 2.8% of reads in the metatranscriptome libraries (Table S1). The unmapped reads were combined and *de novo* assembled using MEGAHIT v1.2.9 (--k-min 23 --k-max 123) (metagenome and metatranscriptome reads were kept separate and assembled separately) [37]. Assembly quality was assessed using QUAST v5.0.2 [38] (Table S2).

### Identification of viral contigs

Viral contigs were identified and annotated using geNomad v1.7.0, which uses a hybrid neural network- and marker-based approach to detect viral sequences in metagenomic data [39]. The metagenomic and metatranscriptomic contigs were piped through geNomad using the default settings to identify putative DNA virus and RNA virus genomic sequences, respectively. A minimum length cutoff of 10 kbp was used for metagenomic contigs [40] and 2 kbp for metatranscriptomic contigs [41] with a minimum viral score of 0.8 for both. Contigs with no viral marker genes predicted by geNomad or with a marker enrichment score < 1.5 were not retained. Contigs with no viral genes called by CheckV v0.8.1 [42] were considered false positives and removed in accordance with the developer recommendations. Filtration removed 397 metagenomic and 516 metatranscriptomic contigs. Contig sequences (including contigs removed during filtration) can be accessed through the associated data repository (https://zenodo.org/records/15281951). Viral contigs were dereplicated at ≥ 99% average nucleotide identity using CD-HIT-EST to cluster any near-identical contigs (no viral contigs clustered together) [43]. Viral genome completeness was assessed with CheckV v0.8.1 using the default settings [42].

### Binning of NCLDV MAGs

Since NCLDVs have characteristically large genomes that may not be contained on a single contig [44, 45], MAGs were generated using a workflow similar to that established by Moniruzzaman et al. for recovering high-confidence NCLDV genomes from metagenomes [46]. Bins were generated with MetaBAT2 v2.15 (-s 100000, -m 10000, -minS 75, -maxEdges 75) using the metagenome contigs and coverage profiles from the metagenome libraries [47]. Bins were screened using ViralRecall v2 (--contiglevel flag) and were retained as putative NCLDV MAGs if the mean ViralRecall contig score was positive [48]. The putative NCLDV MAGs were decontaminated by removing any cellular contigs as described in Moniruzzaman et al. (ViralRecall score < 0 or < 3 hits to the Giant Virus Orthologous Group (GVOG) database) [46]. The putative NCLDV MAGs were further decontaminated by querying translated open reading frames (ORFs) against the complete RefSeq protein database (release 213) using DIAMOND v2.0.14 BLASTP [49]. Contigs were removed if they had zero ORFs with hits of NCLDV origin within the top five hits [46]. NCLDV MAG completeness was estimated using both CheckV [42] and the method described in Schulz et al. [50], which is based on the expected number of conserved nucleocytoplasmic virus orthologous genes (NCVOGs) for a given NCLDV clade.

### Read mapping to viral contigs and NCLDV MAGs

Read mapping was used to determine the distribution of viral contigs and NCLDV MAGs across samples. Reads were mapped using CoverM v0.6.1 with ≥ 90% identity and ≥ 90% read length alignment thresholds [51]. Contig coverage tables were generated by mapping metagenomic reads to DNA virus contigs and metatranscriptomic reads to RNA virus contigs independently. Contig coverage was calculated as the trimmed mean, where values represent the average number of mapped reads to each base-pair after trimming the most deeply and shallowly covered positions (--trim-min 5 and --trim-max 95). Coverage values were then normalized by library size using the calculation described in Emerson et al. [40]. Following benchmarked thresholds [52], a contig was only considered present in a library if ≥ 75% of the contig length was covered by mapped reads in order to account for poorly covered contigs and spurious mappings. Coverage values were reset to zero for contigs with < 75% of the length covered on a per-library basis. Metagenome reads were mapped to RNA virus contigs as a means to assess if any contigs represented endogenized viral elements in cellular genomes. All metagenomic reads, including reads that had mapped to the *Sphagnum* genomes (see above), were mapped to RNA virus contigs using ≥ 90% identity and ≥ 90% read length alignment thresholds.

For the NCLDV MAGs, read mapping and normalization were performed using the same method as above. A MAG was considered present in a metagenome library if reads mapped to ≥10% of the total MAG length, as in Tithi et al. [53]. A MAG was considered transcribed in a sample if ≥ 10% of predicted ORFs had metatranscriptome reads mapped [54].

### Viral contig taxonomic classification and phylogenies

Taxonomic assignments for viral contigs were determined using geNomad v1.7.0 [39]. For the *Caudoviricetes* shared contigs, taxonomy was also assigned using vConTACT2 (Diamond method, ‘ProkaryoticViralRefSeq207-Merged’ database) [55]. For the NCLDV MAGs, the average nucleotide identity (ANI) between the MAGs generated here and a database of available NCLDV genomes was calculated using FastANI v1.33 [56]. A database of 1,177 high-quality NCLDV genomes (isolates and environmental MAGs) compiled by Gilbert et al. [57] was used for the FastANI comparisons. A multi-locus tree was constructed in accordance with the phylogenetic framework presented by Aylward et al. [58]. The concatenated alignment of seven NCLDV marker protein sequences (DNA polymerase B, A32 packaging enzyme, superfamily II helicase, VLTF3 transcription factor, RNA polymerase large subunit, topoisomerase family II, and transcription factor IIB) was generated with ncldv_markersearch v1.1 using the default settings [58]. Gaps in the alignment were trimmed with trimAl v1.2 [59] and the tree was constructed with IQ-TREE v2.2.0.3 (LG + F + I + G4 substitution model with 1,000 ultrafast bootstraps) [60]. For the RNA virus contigs, phylogenetic trees were constructed based on the RNA-dependent RNA polymerase (RdRp) gene. The RdRp amino acid sequences predicted by geNomad were aligned to a custom set of RdRp reference sequences using Clustal Omega v1.2.4 [61] and the trees were constructed using IQ-TREE v2.2.0.3 (-m TEST with 1,000 ultrafast bootstraps ) [62].

### Characterization of the microbial community

DNA-dependent RNA polymerase was used as a marker for prokaryotic (RpoB) and eukaryotic (Rpb1) genomes [63, 64]. Contigs containing RpoB/RPB1-like sequences were retrieved from the metagenome coassembly by DIAMOND v2.0.14 (BLASTX, e-value < 1e-5) using a database of RpoB (TIGR02013, TIGR03670) and RPB1 (CD02733, CD02584) protein sequences. To confirm the presence of RpoB/RPB1, proteins were predicted on metagenomic contigs using Prodigal v2.6.3 [65] and annotated with eggNOG-mapper [66] and the eggNOG v5.0 database [67]. The top BLASTP hit (*i*.*e*., lowest e-value) for the RpoB/RPB1 gene was used for taxonomic assignment. Reads were mapped to RpoB/RPB1 using CoverM v0.6.1 with ≥ 90% identity and ≥ 90% read length alignment thresholds [51].

## Results

### Microbial community structure and core taxa

We first assessed microbial community structure in the *Sphagnum* samples. Prokaryotic community composition in the metagenomes was similar to what has previously been described using 16S rRNA gene sequencing approaches [12, 32, 68]. Prokaryotic communities were consistently dominated by Proteobacteria based on both observed richness (54% of RpoB-containing contigs) and relative abundance in the metagenomes (63.5% ± 1.4%, mean relative read abundance ± SE), followed by Acidobacteria (13% of RpoB contigs, 15.5% ± 0.8%) (Figure S1, Table S3). The community structure of the transcriptionally active community reflected the metagenome structure, where Proteobacteria generally dominated the pool of mapped RpoB transcripts (42.7% ± 2.5%) (Figure S1). Cyanobacteria displayed larger representation in the metatranscriptomes (24.9% ± 19.3%) relative to the corresponding metagenomes, a trend that has been observed previously [32]. A shared prokaryotic community comprised largely of Proteobacteria (125 RpoB-containing contigs) and Acidobacteria (27 contigs) was detected in all eight metagenomes by read mapping (Figure S2, Table S3). The majority of the shared RpoB were also detected in the metatranscriptomes and considered transcriptionally active (160 of 171 contigs) (Table S3). Genera within the phyla Alphaproteobacteria and Acidobacteria have been found to dominate the North American *Sphagnum* core microbiome [32]. We detected related genera in the core based on sequence similarity searches and RpoB phylogenetic placement, including *Acidocella*, *Acidisphaera*, and *Acidisoma* (Alphaproteobacteria), as well as *Granulicella* (Acidobacteria). Shared taxa also included diazotrophic methanotrophs within the family *Beijerinckiaceae* (phylum Alphaproteobacteria) (Table S3, Figure S2).

Eukaryotic communities were dominated by phototrophic taxa, including *Chlorophyta* (green algae, 35.2% ± 5.9% of RPB1 relative abundance) and *Bacillariophyta* (diatoms, 27.7% ± 7.0%). *Chrysophyceae* (golden algae, 9.3% ± 3.0%), *Cryptophyta* (cryptomonads, 3.2% ± 1.5%) (Figure S1, Table S3), Ascomycota (fungi, 8.8% ± 2.4%) and Evosea (4.8% ± 2.5%) sequences were also detected. *Chlorophyta* and Ascomycota were collectively detected in every metagenome, while *Bacillariophyta*, *Chrysophyceae*, Euglenozoa, and Evosea were detected in seven of the eight (Figure S1). Only one RPB1 contig (*Chlorophyta*) was detected in all eight metagenomes (Table S3). The composition of shared micro-eukaryotic assemblages in *Sphagnum* are not well defined, but *Chlorophyta*, *Chrysophyceae*, and Ascomycota fungi have been documented as common constituents of *Sphagnum* microbiomes sampled across various climates [13].

### Taxonomic diversity and distribution of viral contigs

**Fig. 1:**
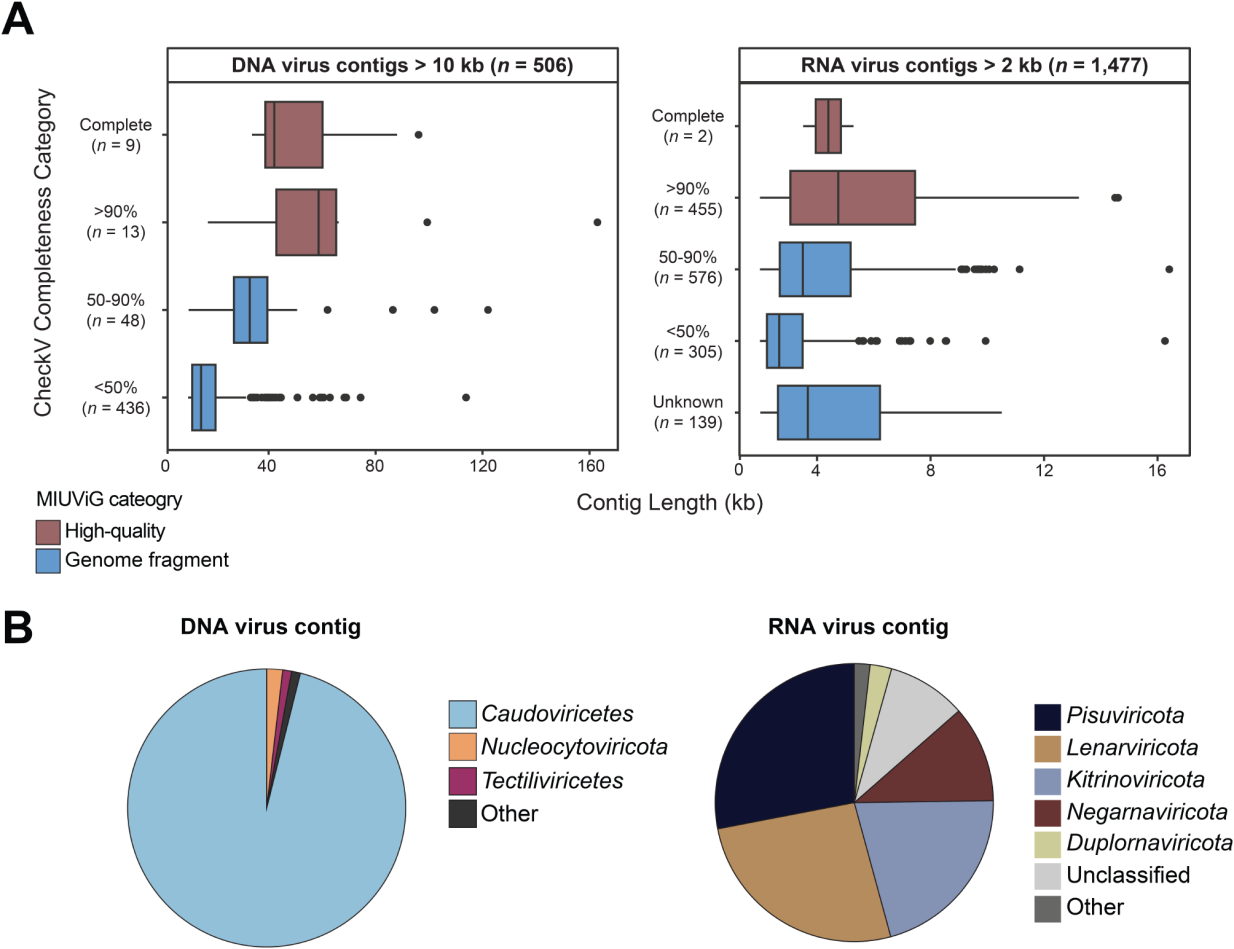
Overview of the quality and taxonomic classification of the DNA virus and RNA virus contigs. **(A)** Contigs are grouped by CheckV completeness category: low-quality (< 50% complete), medium-quality (50–90%), high-quality (> 90%), and complete. Groups classified as high-quality draft genomes by MIUViG standards (> 90%) are shown in red. **(B)** Proportion of viral taxonomic groups based on geNomad-assigned taxonomy for all DNA virus and RNA virus contigs detected. ‘Other’ DNA virus taxa include: *Maveriviricetes*, *Polintoviricetes*, *Naldaviricetes*, unclassified *Bamfordvirae*, and unclassified virus. ‘Other’ RNA virus taxa include: *Permutotetraviridae* and *Artverviricota*

We screened the metagenome and metatranscriptome contigs to identify virus-like sequences and used read mapping to assess their distribution across the eight *Sphagnum* samples. We recovered 506 DNA virus-like and 1,477 RNA virus-like genomic sequences (virus contigs) from the metagenome and metatranscriptome co-assembly, respectively (Fig. 1, Table S4-S5). Using a minimum length cutoff of 10 kb for DNA viruses and 2 kb for RNA viruses, the mean sequence length was 21.9 kb for DNA virus and 4.4 kb for RNA virus contigs. As is common with the identification of viral genomes from short-read assembled contigs [69], the majority of viral sequences were categorized as genome fragments based on the Minimum Information about an Uncultivated Virus Genome (MIUViG) standards (Fig. 1A). Approximately 4% of DNA virus contigs (*n* = 22) and 30% of RNA virus contigs (*n* = 457) represented high-quality viral genomes (Fig. 1A).

The majority of the DNA virus contigs were classified by geNomad as *Caudoviricetes* (tailed dsDNA phage, 487 contigs), in addition to *Tectiliviricetes* (includes the tail-less dsDNA phage, 5 contigs) and *Nucleocytoviricota* (NCLDV, 5 contigs) (Fig. 1B, Table S5). Contigs classified as *Maveriviricetes* (virophage, 1 contig), which obligately co-infect with NCLDV, and *Polintoviricetes* (polinton-like viruses, 4 contigs) were also identified. Regarding the RNA virus (*Riboviria*) contigs, all five phyla within the kingdom *Orthornavirae* (RdRp-encoding viruses, *n* = 1,453) were identified as well as members of the *Pararnavirae* (reverse-transcriptase-encoding viruses, *n* = 3) (Table S5). Overall, most RNA virus contigs were classified into the *Pisuviricota*, *Lenarviricota*, and *Kitrinoviricota* phyla that contain positive-sense single-stranded as well as double-stranded RNA viruses (Fig. 1B). The orders with the most representation included the *Narnaviridae* (*n* = 151), *Mitoviridae* (*n* = 89), and *Picornaviridae* (*n* = 138), as well as orders of plant viruses such as *Potyviridae* (*n* = 104) and *Tombusviridae* (*n* = 138). Hereon, we refer to the “virome” as meaning the set of viral sequences identified from the metagenomes.

**Fig. 2:**
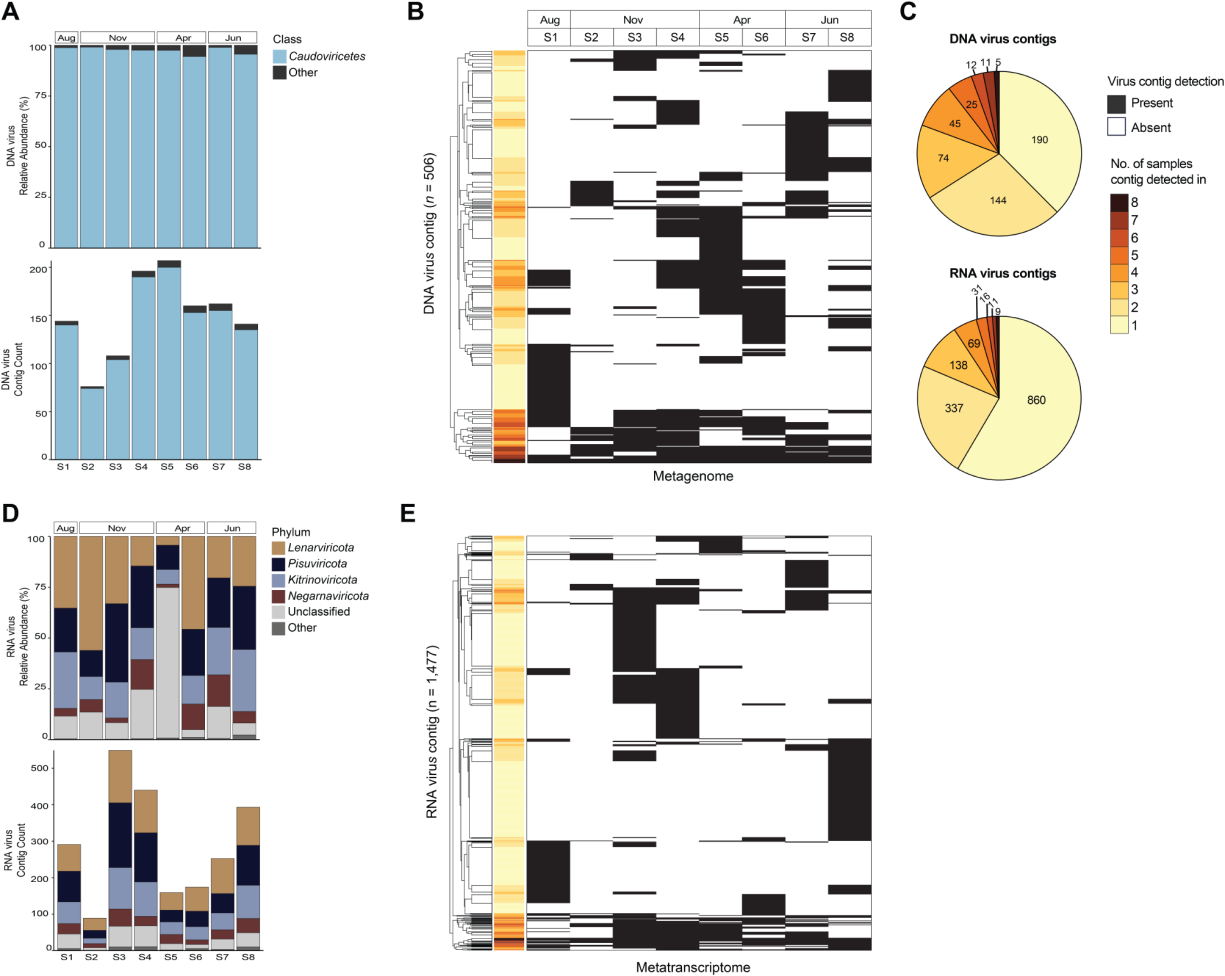
**(A)** Relative abundance and proportion of *Caudoviricetes* contigs in the metagenomes. **(B)** Distribution (presence-absence) of the DNA virus contigs in the metagenomes. Each row represents a contig. Average linkage hierarchical clustering was applied based on contig presence-absence. Color-coded rows indicate the number of metagenomes each contig was detected in. **(C)** Pie chart shows the total number of contigs within each detection category (i.e., the number of samples a contig was detected in) (4 RNA contigs were not detected in any metatranscriptomes). **(D)** Relative abundance and proportion of RNA virus contigs in the metatranscriptomes. **(E)** Distribution of the RNA virus contigs in the metatranscriptomes

Phage-like contigs were a core component of the *Sphagnum* DNA virome, where *Caudoviricetes* dominated the set of viral contigs in every sample both in terms of count and relative abundance (Fig. 2A). However, viral community composition varied between the samples at the contig level. The distribution of viral contigs in the metagenomes was generally patchy (Fig. 2B), and Bray-Curtis dissimilarity indices ranged from 0.42 to 0.90 between samples when calculated based on viral contig presence-absence (Table S6). Furthermore, nearly 40% of all DNA virus contigs were detected in only a single sample (Fig. 2C). RNA virus contigs displayed a similar pattern in taxonomic and contig-level distribution across samples. Within samples, most contigs were assigned to the RNA virus phyla *Lenarviricota* (25–39% of contigs), *Pisuviricota* (20–33% of contigs), and *Kitrinoviricota* (17–24% of contigs) (Fig. 2D). Between samples, dissimilarity indices ranged from 0.61 to 0.95 based on contig presence-absence and the majority of RNA virus contigs (~ 51%) were only present in a single sample (Fig. 2E, Table S6).

We observed virus contig sharing between plants when grouped by the month sampled (Figure S3), however, given our small sample size we focused on contigs that were shared between individual plants across all months sampled. In total, five DNA virus and nine RNA virus contigs were detected in every sample and were further characterized as possible members of the *Sphagnum* core virome (Fig. 2C). For brevity we refer to the viral contigs detected in every sample as “shared” contigs. The shared contigs made up less than 1% of the total number of contigs identified, and they did not disproportionally comprise the virome relative to the other DNA and RNA virus contigs based on their relative abundance (Figure S4).

### Characterization of the shared DNA virus contigs

**Fig. 3:**
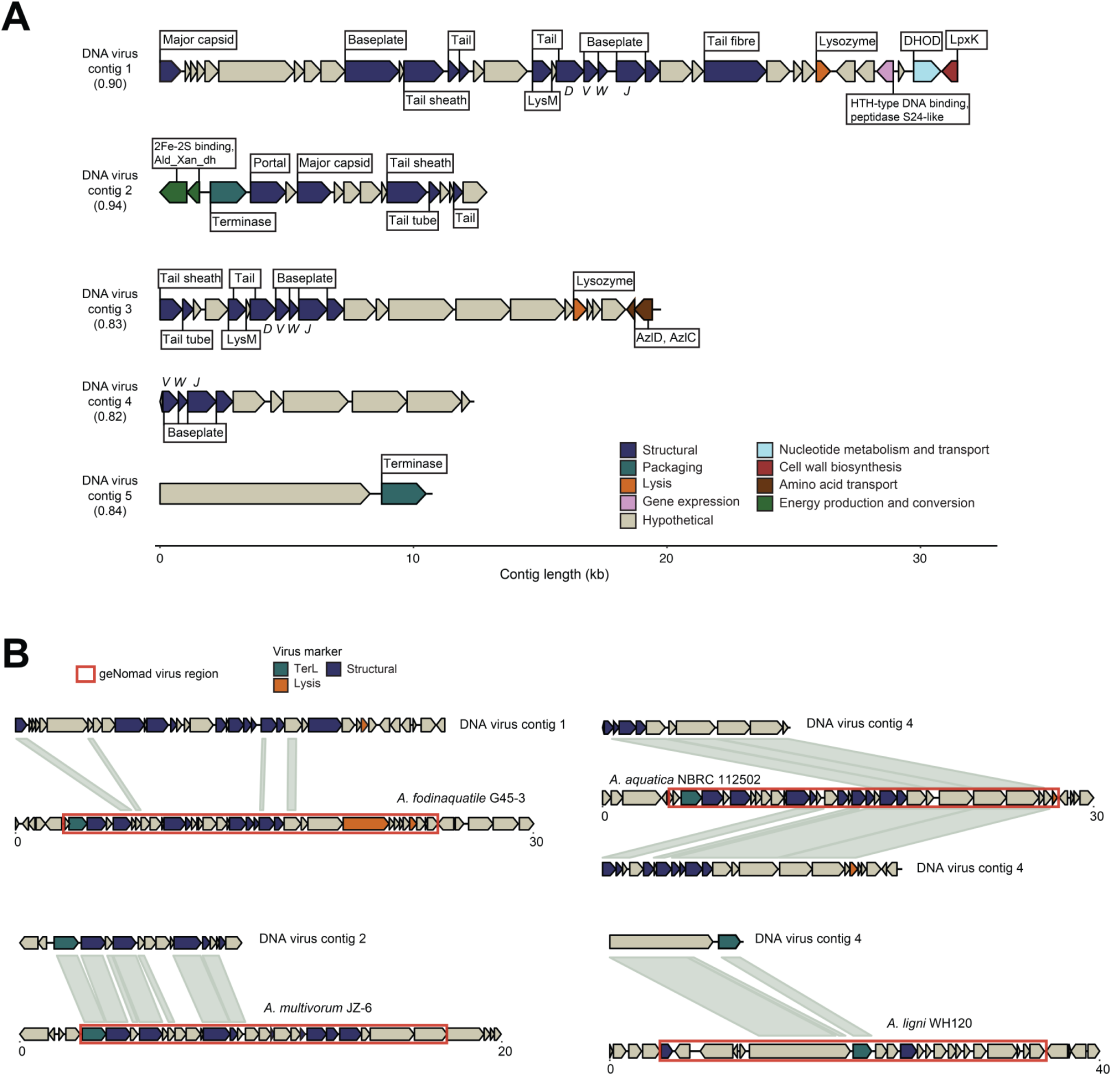
**(A)** Predicted ORFs and annotations for the core DNA virus contigs. The value in parentheses represents the geNomad virus score. Annotation details are available in Table S7. All contig illustrations were made using the gggenomes R package. **(B)** Nucleotide alignment (BLASTN) of the core metagenomic contigs to RefSeq non-viral genomes. The viral region predicted by geNomad is outlined in orange. Details regarding the BLASTN results and prophage region coordinates are available in Table S10

We examined the gene content, taxonomic classification, and possible replication styles of the shared viral contigs. All five of the shared *Caudoviricetes* contigs were categorized as genome fragments by MIUViG standards. Indeed, no individual contig encoded all of the genomic modules that are generally present in sequenced phage genomes and required for replication (*i*.*e*., the DNA replication, structural, packaging, and lysis modules) (Fig. 3, Table S7) [70, 71]. Furthermore, no shared contigs contained any predicted gene products with sequence similarity to those that would putatively be involved in phage DNA replication mechanisms (*e*.*g*., initiators, helicases/primases, DNA polymerases) [72]. Collectively the contigs were predicted to encode genes that may belong to structural and packaging modules, as well as genes potentially involved in cell lysis and gene expression (Fig. 3A). Although gene content predictions were similar among the shared phage-like contigs, most contigs had little sequence similarity to one another at the nucleotide level (0–11% contig coverage). Two contigs (DNA Virus Contigs 3 and 4) did display a higher degree of overlap (58% and 93% coverage, respectively), but with < 70% nucleotide identity and were therefore treated as distinct sequences (Figure S5, Table S8).

We examined shared protein content between the contigs and RefSeq viral genomes using vConTACT2 to provide additional taxonomic resolution [55]. Three of the five contigs (DNA Virus Contig 1, 3, and 4) were placed in a viral cluster with Ralstonia phage Raharianne, a strain of lytic *Rahariannevirus* phages that were isolated from the plant pathogenic bacterium *Ralstonia solanacearum* (order *Burkholderiales*) [73]. The two remaining contigs did not cluster with any RefSeq viral genomes, but an additional contig (DNA Virus Contig 2) displayed homology to *Rahariannevirus* protein sequences, namely putative structural genes (Figure S6, Table S9).

### Shared phage-like contigs exhibit similarities to temperate phage and potentially degraded prophage in bacterial genomes

Features of the shared contigs suggested they may represent fragmented sequences of temperate phage, phage genomes integrated into host genomes (prophage), or non-inducible remnants of integrated phage (degraded or cryptic phage). Prophage can be predicted from metagenomic contigs by identifying putative microbial genes flanking viral regions [42]. Three core contigs (DNA Virus Contig 1, 2, and 3) were categorized as prophage by CheckV and contained predicted metabolic genes that flanked virus-specific structural genes on one side of the contig (although we do not rule out that these could be phage-encoded accessory metabolic genes) (Fig. 3A). The putative host-encoded genes included those involved in pyrimidine biosynthesis (DHOD), amino acid transport (AzlCD), energy production and conversion (subunits of Aldehyde, CO, or xanthine dehydrogenase), and lipid A biosynthesis (LpxK) (Fig. 3A).

None of the core contigs contained a putative complete lysogeny control module (based on the lack of hits to integrases/recombinases, excisionases, transposases, or repressor/antirepressor gene products), but one contig (DNA Virus Contig 1) contained a putative S24-type peptidase that may function in the microbial SOS response or as a phage lytic cycle repressor. Additionally, four contigs (DNA Virus Contig 1 through 4) contained putative baseplate domains with homology to those conserved in P2-like and Mu-like temperate phages (gpVWJ domains) [74, 75].

Lastly, the majority of the predicted gene products on all five contigs had best BLASTP hits to genes annotated on bacterial genomes (Table S7). We subsequently investigated if the shared phage-like contigs aligned to prophage or cryptic phage regions within RefSeq bacterial genomes using BLASTN. Four contigs (DNA Virus Contig 1 through 4) aligned to single continuous regions within *Acetobacteraceae* genomes (phylum Alphaproteobacteria), where for three of which the majority of the contig aligned at > 65% percent nucleotide identity (56–93% contig coverage) (Fig. 3B, Table S10). DNA Virus Contig 1, the longest contig, had relatively low coverage (10%) at ~ 73% nucleic acid identity. The final and shortest contig (DNA Virus Contig 5) aligned to a region of an *Acidobacteriaceae* genome (phylum Acidobacteria) with ~ 75% nucleic acid identity and 78% contig coverage (Fig. 3B, Table S10).

Using the top BLASTN RefSeq genome hit as a case study, all of the RefSeq genomes (either complete genomes or scaffolds) had viral regions identified by geNomad that corresponded to the *Sphagnum* viral contig alignment region (Fig. 3B). The predicted viral regions within the bacterial genomes contained putative phage structural, DNA packaging, and lysis genes (Figure S7). Aside from the shortest contig that contained only two predicted ORFs (DNA Virus Contig 5), the core contigs were similar in length to the prophage region (Fig. 3B). None of the prophage regions had the gene modules generally necessary for prophage excision and replication and may represent degraded prophage or phage-derived elements.

### Phylogenomics and distribution of NCLDV MAGs

As noted previously, five metagenomic viral contigs were classified as *Nucleocytoviricota*. The length of each contig was less than 50 Kbp and no contigs were detected in all eight metagenomes (Table S4). Metagenome-assembled genomes (MAGs) were generated to further investigate shared NCLDV populations to account for the large size of NCLDV genomes [44, 45]. Three NCLDV MAGs were generated from the *Sphagnum* metagenomes, each larger than 100 Kbp and contained less than 15 contigs (Table 1). The MAGs were categorized as genome fragments by MIUViG standards and ranged from 42 to 65% complete based on the presence of conserved marker genes (NCVOGs). Further details regarding the completeness and quality of each NCLDV MAG are provided in Table S11.

**Table 1:** Overview of the NCLDV MAG quality and phylogenetic placement

NCLDV MAG	Length (bp)	No. contigs	No. ORFs	Completeness (%)	Order
MAG-1	217,151	11	260	Low (43)	*Pimascovirales*
MAG-2	122,196	1	127	Medium (65)	*Pimascovirales*
MAG-3	165,244	8	167	Low (42)	*Asfuvirales*

**Fig. 4:**
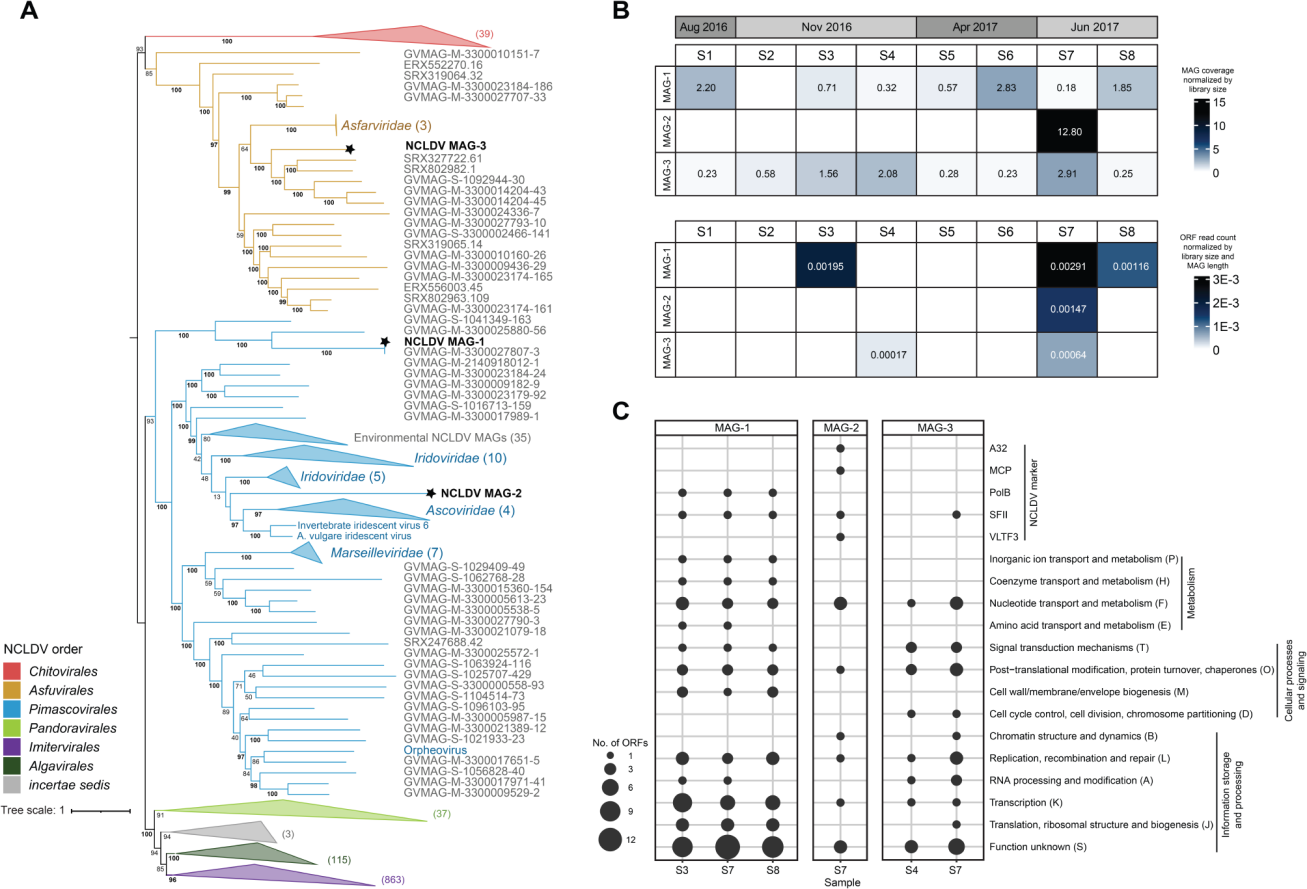
**(A)** Phylogenomic analysis of the NCLDV MAGs. The MAGs generated in this study are denoted by stars. Some lineages are collapsed for clarity and the values in parentheses represent the number of collapsed nodes. The tree is rooted at the *Pokkesviricetes* (includes *Chitovirales* and *Asfuvirales*). Nodes with labels beginning with GVMAG and ERX/SRX represent environmental MAGs generated from Schulz et al. [50] and Moniruzzaman et al. [46], respectively. Bootstraps ≥95% are shown in bold. **(B)** Heatmap illustrating the distribution of NCLDV MAGs in the metagenomes (top panel) and metatranscriptomes (bottom panel). **(C)** Number of ORFs detected in the metatranscriptomes grouped by COG functional category or NCLDV marker. Only samples where at least one MAG was transcriptionally active are shown

The NCLDV MAGs were most closely related to viruses belonging to the orders *Pimascovirales* and *Asfuvirales* (Fig. 4A). NCLDV MAG-3 clustered with environmental *Asfuvirales*-like MAGs related to the cultivated *Asfarviridae*, represented here by African swine fever virus (ASFV) (Fig. 4A). Based on protein sequence similarity (BLASTP), MAG-3 is likely related to Pacmanvirus A32, an asfarvirus relative isolated from the amoeba *Acanthamoeba castellanii* [76] (Table S12). MAG-1 fell within a sister clade to the *Pimascovirales* that contained no cultivated NCLDVs (Fig. 4A). Notably, MAG-1 was closely related to and displayed 98% ANI with a reference NCLDV MAG (MAG reference ID: GVMAG-M-3300027807-3) (Fig. 4A). This reference MAG was generated from a *Sphagnum* metagenome collected in 2015 from the same site sampled in this study (IMG Taxon ID 3300027807) [50]. Lastly, MAG-2 was most closely related to viruses of the families *Iridoviridae* and *Ascoviridae* (order *Pimascovirales*) (Fig. 4A). Thirteen of the 18 MAG-2 proteins with top hits to RefSeq viruses were *Iridoviridae* sequences, with BLASTP scores ranging from 24 to 46% percent amino acid identity, further suggesting MAG-2 is a relative of the iridoviruses (Table S12).

Read mapping analysis showed that MAG-1 and MAG-3 were detected in all eight and in seven of the eight metagenomes, respectively, whereas MAG-2 was detected in only a single sample (Fig. 4B, Table S13). All three MAGs were transcriptionally active in at least one sample (*i*.*e*., they passed the detection threshold where > 10% of ORFs had at least one metatranscriptome read mapped), where MAGs ranged from 32 to 50% of predicted ORFs with reads mapped within a sample (Fig. 4B, Table S13). MAG-2 and MAG-3 were transcriptionally active in two and three samples, respectively. The majority of putative transcribed genes were of unknown function, but also included genes that may be involved in information storage and processing, cellular processes and signaling, and metabolism (Fig. 4C, Table S14). Various NCLDV marker genes were also detected in the metatranscriptomes, including the packaging ATPase (A32), major capsid protein (MCP), DNA polymerase family B (PolB), DEAD/SNF-2 helicase (SFII), and poxvirus late transcription factor (VLTF3) (Fig. 4C, Table S14).

Notably, horizontally acquired NCLDV-like regions have been found within the genome of the moss species *Physcomitrium patens*, including homologues to *Pimascovirales* genes [77]. We performed a phylogenetic analysis of the DNA polymerase marker gene to examine the relationship between the *Sphagnum*-associated NCLDV MAGs, extant NCLDV lineages, and the NCLDV-like regions integrated within the *P*. *patens* genome. While MAG-1 and MAG-2 were related to the *P*. *patens* NCLDV remnants, they were placed within the clade of extant *Pimascovirales* (Figure S8).

### Phylogenetic analysis of the shared RNA virus contigs

Five of the nine shared RNA virus contigs were determined to be high-quality and all but one contig (RNA Virus Contig 1) encoded an RNA-dependent RNA polymerase (RdRp) with the conserved ABC motifs that are involved in catalysis [78] (Table S5). Additionally, no contigs recruited any metagenome reads, which otherwise may have suggested that they were assembled from transcribed endogenized viral elements in cellular genomes. The shared virus contigs were broadly classified as *Lenarviricota* (5 contigs), *Kitrinoviricota* (1 contig), and *Pisuviricota* (3 contigs) by geNomad’s marker gene-based taxonomic assignment (Table S5), and generally displayed low levels of amino acid similarity to known sequences (Table S15). We constructed phylogenies of the conserved RdRp gene to better asses the relationship of these RNA virus-like contigs to known viruses.

**Fig. 5:**
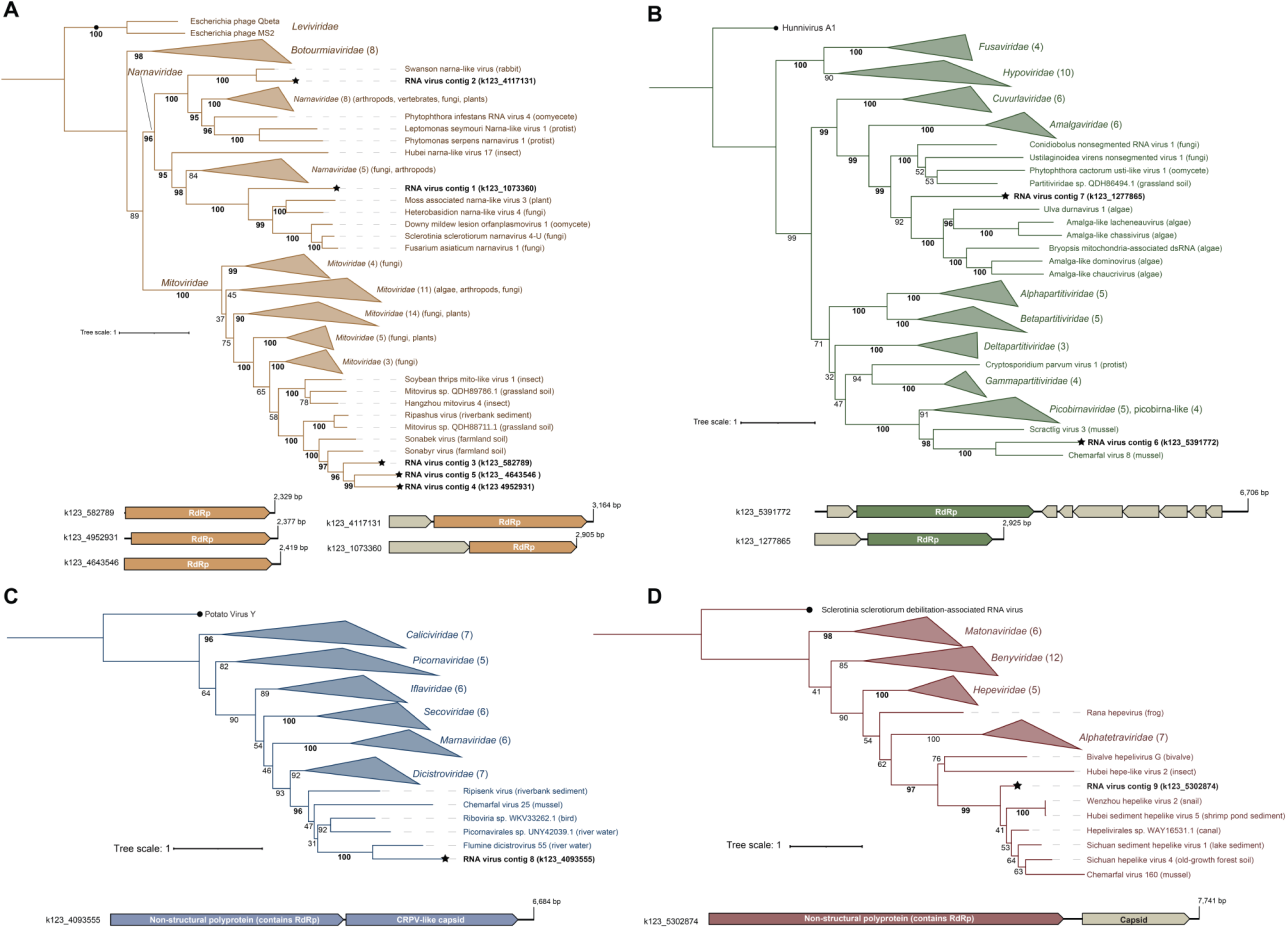
RdRp phylogenetic placement and genome organization of the nine putative RNA viruses detected in all eight samples. **(A)***Lenarviricota* (VT + F + I + G4 best fit substitution model). **(B)***Durnavirales* (Blosum62 + F + I + G4). **(C)***Picornavirales* (Q.pfam + F + I + G4). **(D)***Hepelivirales* (Q.pfam + F + I + G4). The core RNA viruses are labeled in bold and denoted by stars. Bootstrap values ≥95 are emphasized in bold. Trees are rooted at the outgroup (denoted by circle). Some lineages are collapsed for clarity and the values in parentheses represent the number of collapsed nodes. The host or isolation source is shown in parentheses. Only the RdRp domain was used for phylogenetic analysis for the *Picornavirales* and *Hepelivirales*. The full trees are available in Figure S9 within Supplementary Material 1

Five shared contigs fell within the *Mitoviridae* and *Narnaviridae* families (phylum *Lenarviricota*) (Fig. 5A), which contain capsid-less + ssRNA viruses that typically encode only a RdRp gene. Three contigs (RNA Virus Contig 3 through 5) clustered with viral-like sequences recovered from soil and sediment metatranscriptomes (Fig. 5A). While the three *Mitoviridae* contigs discovered here were closely related, they displayed on average 38% RdRp amino acid identity to one another and thus may represent separate species by ICTV standards (Table S16) [79]. RNA Virus Contig 1 clustered with narnaviruses of plants, fungi and oomycetes, and showed 25% RdRp amino acid identity with an oomycete-associated virus (Downy mildew lesion associated orfanplasmovirus 1). RNA Virus Contig 2 had 51.13% RdRp amino acid identity to and clustered with a vertebrate-associated narna-like virus (Swanson narna-like virus). Typical of the *Mitoviridae* and *Narnaviridae*, none of the shared mitovirus- and narnavirus-like contigs encoded identifiable capsid genes (Fig. 5A).

RNA Virus Contigs 6 through 8 were assigned to the *Pisuviricota* (Table S3, Fig. 5B-C). RNA Virus Contig 6 displayed 23.08–28.29% RdRp protein identity to aquatic invertebrate-associated and soil picobirna-like viruses, and it clustered with *Picobirnaviridae* and picobirna-like + dsRNA viruses (Fig. 5B, Table S15). RNA Virus Contig 6 likely represents a chimeric contig since the contig length and number of predicted ORFs was irregular for picobirna-like genomes (Fig. 5B). Further details regarding this contig are provided in Supplementary Material 1. RNA Virus Contig 7 clustered with members of a proposed algae-infecting clade of + dsRNA *Partitiviridae* relatives (partiti-like viruses) [80], and had 29.01% RdRp protein identity to the algae-associated Amalga-like dominovirus. The partitivirus-like contig did not encoded a recognizable coat protein (Fig. 5B). *Partitiviridae* spp. can have segmented genomes where the capsid and RdRp are on separate segments and may therefore be missed here, though the partitivirus-like relatives of RNA Virus Contig 7 are nonsegmented [80, 81].

RNA Virus Contig 8 clustered with the *Dicistroviridae* and dicistro-like viruses on the phylogeny (Fig. 5C), and it showed 25.04–34.55% RdRp amino acid identity to sequences from invertebrates and aquatic sources (Fig. 5C). Lastly, RNA Virus Contig 9 had top BLASTP hits (38.83–51.98% RdRp protein identity) to and clustered with *Hepelivirales*-like viruses associated with invertebrates and from various environmental sources (Fig. 5D). Both the dicistrovirus- and hepevirus-like contigs encoded putative capsid proteins in addition to the RdRp (Fig. 5C and D).

## Discussion

Tissue samples from individual plants collected over a ten-month period enabled us to screen for the presence of shared viral elements associated with *Sphagnum*. Our paired metagenomic and metatranscriptomic approach identified shared viral contigs that may have originated from phage, NCLDV, and RNA viruses, suggesting a diverse and previously neglected viral component of the *Sphagnum* core microbiome. The shared contigs described here are likely not a definitive catalogue, both due to our conservative viral identification methods and the challenges in recovering viral sequences from bulk metagenomes [82, 83]. Additionally, with our limited number of samples it is difficult to assess to what extent environmental factors, such as season or geographic proximity, explain virome composition. For example, the seasonal succession of viral communities in the surface peat of *Sphagnum*-dominated peatlands has been observed but is currently unexplored in communities associated with the living plant tissue [84]. Future work incorporating more *Sphagnum* samples, a larger geographic area, and longer timeframes may illuminate both how widespread the viral elements identified here are and the influence of environmental variables. Nonetheless, the observation that viral contigs were shared between *Sphagnum* sampled from different months and micro-topographies suggests that they may be a common component of the virome despite these environmental differences.

The *Sphagnum* core microbiome includes bacterial symbionts that are implicated in plant health and in peatland nutrient cycling [32], yet it is unknown if phage infection modulates moss-bacteria interactions. The shared phage-like contigs identified here shared predicted gene content with lytic phages of the phytopathogen *Ralstonia solanacearum* (order *Burkholderiales*) [73]. To our knowledge, no phytopathogenic *Burkholderiales* have been described in association with peat mosses, but other members are commonly associated with *Sphagnum* and may suppress pathogens, produce plant growth-promoting hormones, and supply nutrients [32, 85, 86]. Although *Burkholderiales* phages could therefore have indirect effects on moss growth, we do not presume the shared phage-like contigs represent *Burkholderiales* phages or remnants of *Burkholderiales* phages based solely on their estimated taxonomy, especially since these contigs are fragmented.

Additionally, features of the shared phage-like contigs suggested they may represent temperate phage or regions in bacterial genomes that were derived from defective prophage. These features included the presence of flanking metabolic genes, conserved temperate phage baseplate motifs, and the presence of highly similar regions in sequenced *Acetobacteraceae* and *Acidobacteriaceae* genomes. For four of the five contigs in particular, the majority of the contig aligned to the reference bacterial genomes. Genera within the *Acetobacteraceae* (*Acidocella*, *Acidisphaera*, and *Acidisoma*) and *Acidobacteriaceae* (*Granulicella*) are dominant members of the core *Sphagnum* microbiome [32], and markers assigned to these families were shared between all eight samples examined in this study as well. While not examined in peat moss-associated bacteria specifically, prophage regions have been reported in *Acetobacteraceae* genomes [87] and Acidobacteria harbor a recognized phage mobilome that is implicated in host survival in harsh environments [88, 89]. In addition, prophage and cryptic phage have the ability to impact host cell physiology as well as provide protection against phage infection [90, 91], and vertically inherited cryptic phage can give rise to systems co-opted by bacteria, such as type VI secretion systems, tailocins, and gene transfer agents [75, 92].

While the origin and function of the shared phage-like contigs is uncertain, the similarity between the viral contigs and RefSeq bacterial genomes suggests a gene sharing network between bacteria and phage in the peat moss microbiome. Similarly, Bragina et al. observed that *Sphagnum* metagenomes were enriched in mobile genetic elements, including temperate phage genes, and posited that the peat moss microbiome may be highly plastic in part due to phage-mediated gene transfer [12]. Future studies are warranted to unravel the prevalence of both functional phage and phage-derived elements in moss-associated symbionts since there is the potential for phage-bacteria interactions to influence the evolution and plasticity of the *Sphagnum* core microbiome. A combination of sequencing the genomes of bacteria isolated from peat moss, long-read bulk metagenomic sequencing, and sequencing of the viral fraction (DNA viromics) may provide greater insight into phage dynamics and phage-mediated gene transfer events in the *Sphagnum* microbiome.

Nucleo-cytoplasmic large DNA viruses are a diverse group of eukaryotic viruses that have the potential to modulate microbial community structure and biogeochemical cycles by altering infected cell metabolism and by lysing their hosts [46, 50]. Little is known regarding the ecology of NCLDV in peatlands aside from the detection of putative NCLDV transcripts in *Sphagnum* metatranscriptomes [30]. Our analysis identified NCLDV MAGs related to the *Pimascovirales* and *Asfuvirales* that may represent common members of the *Sphagnum* microbiome. Asfarvirus-like transcripts have been reported in Minnesotan *Sphagnum* metatranscriptomes [30], further suggesting that asfarvirus-like NCLDV populations are persistent and actively replicating in the peat moss microbiome. While the cultivated *Asfarviridae* were isolated from pigs, recent work has expanded the known host range of asfarviruses to include unicellular eukaryotes [93, 94], and our sequence similarity searches linked the asfarvirus-like MAG to related viruses of amoeba. Furthermore, the detection of a divergent *Pimascovirales* lineage in nearly all of the metagenomes combined with the assembly of a highly similar MAG from a separate study provides evidence that this divergent *Pimascovirales* lineage may also represent a viral constituent of the peat moss core virome. *Pimascovirales* spp. infect a broad range of organisms, including animals (invertebrates and vertebrates) and amoeba, and their detection in marine metagenomes suggests the order may also include viruses of other undetermined protists [95–97]. If these NCLDV lineages infect protistan guilds they could have impacts on the *Sphagnum* microbiome as both grazing and non-grazing protists influence microbial structure and nutrient cycling in plant-associated and peat communities (*e*.*g* [98, 99]).

As noted earlier, *Pimascovirales* homologues have been discovered within the genome of *P. patens*, a moss species related to the *Sphagnales* [77]. Remnants of NCLDV genomes within *P*. *patens* suggests that they were horizontally acquired from the ancestors of this NCLDV order, and NCLDV were therefore present in the ancestral *P*. *patens* microbiome. These remnant NCLDV-like genes are transcriptionally silenced in *P*. *patens* [77], but some loci showed evidence of expression and may play roles in protecting moss gametes from viral infection [100]. Our findings indicated that the NCLDV MAGs were transcriptionally active, including the transcription of viral marker genes that would be utilized during viral replication, and that they were more closely related extant viral lineages. Together this suggested that these MAGS are representative of contemporary NCLDV lineages as opposed to endogenized sequences in non-viral genomes. The *Pimascovirales*-like MAGs may represent extant relatives of an ancestral NCLDV lineage that has long been associated with mosses. Future work is required to determine if there is evidence of past NCLDV-*Sphagnum* interactions as is the case for *P*. *patens*.

RNA viruses in plant viromes can have harmful, negligible, or even beneficial effects on the host plant [101], and may have indirect impacts on plant health by infecting members of the microbiome. Phylogenetic affinities and best BLAST hits indicated that the shared RNA virus contigs included mitovirus-like (*n* = 3) and narnavirus-like viruses (*n* = 2). These could represent ssRNA viruses of fungi in the microbiome as many known *Mitoviridae* and *Narnaviridae* spp. infect fungi and since Ascomycota markers were detected in all eight metagenomes [102]. Fungal viruses may be of interest as fungi can act both as parasites and beneficial symbionts of bryophytes (i.e., non-vascular land plants, including mosses) [11, 103]. We note that while *Mitoviridae* and *Narnaviridae* spp. were once thought to exclusively infect fungi, deep sequencing approaches have revealed related sequences that may infect unicellular algae, vascular plants, and invertebrates [104–106]. Therefore, additional work is required to determine the host range of these putative viruses.

One shared RNA virus contig was partitivirus-like. *Partitiviridae* spp. infect plants, fungi, and protozoa [107]. Although bryophyte viromes are under-sampled, partitivirus- and amalgavirus-like sequences have been found in bryophyte transcriptomes and are thought to infect mosses [108, 109]. The contig identified here was similar to members of a recently proposed green algae-infecting clade of dsRNA partiti-like viruses [80]. This could indicate that partiti-like viruses of algae are common in the *Sphagnum* microbiome, but this requires validation. Furthermore, we detected shared contigs that were hepevirus-like (*n* = 1) and dicistrovirus-like (*n* = 1). Both *Hepeviridae* spp. and *Dicistroviridae* spp. infect animals (vertebrates and insects, respectively), but notably hepevirus-like RdRp sequences have been detected in a *Sphagnum* transcriptome [108].

Future efforts may aim to unravel if the shared RNA viruses described here infect members of the *Sphagnum* microbiome (e.g., fungi, algae, or other eukaryotes) or *Sphagnum* itself. Mining *Sphagnum* transcriptomes alongside bulk metatranscriptome analysis could help to differentiate viruses of the host plant versus of the microbiome that is otherwise difficult to determine from bulk metagenomes and phylogenetic approaches alone. Validating the hosts of these putative shared RNA viruses will be crucial for understanding any ecological implications or effects on plant health. Additionally, while not examined in detail in this work, we detected viral contigs assigned to orders of plant-infecting RNA viruses, such as *Tombusviridae* and *Potyviridae*, so *Sphagnum* metatranscriptomes may be useful resource for discovering moss RNA viruses.

The presence of shared RNA viruses related to those that generally have no extracellular stage of replication (i.e., the *Mitoviridae*, *Narnaviridae*, and *Partitiviridae*) invites additional questions regarding the modes of acquisition and transmission of viruses within the peat moss core microbiome. Shared RNA viruses may be maintained in the core virome through persistent, and perhaps asymptomatic, infection with eukaryotic taxa in the core microbiome or the host plant, which can be a common occurrence with some of these RNA virus orders [110, 111]. Similarly, prophage or cryptic phage elements may have be detected in all of the metagenomes because they are present in the genomes of core bacterial taxa, in this case *Acetobacteraceae* and *Acidobacteriaceae* symbionts, and are reflective of the presence of the host organism. Furthermore, some common bacterial symbionts can be transmitted vertically or be acquired from the surrounding peat and water [31, 68], and this may be a mechanism of transmission for strictly intracellular viruses or viral elements. Possible modes of transmission and maintenance of viruses in the *Sphagnum* virome requires further study.

It is worth noting that contaminant viruses can be present in sequencing reagents [112, 113]. None of the shared DNA or RNA viral contigs discussed here were classified as viral groups that have been detected in metatranscriptome sequencing reagents (e.g., *Tombusviridae*, *Totiviridae*, and *Lentiviridae*) [113]. We note that in human samples, iridovirus-like reads were associated with a component of RNA sequencing methods (RNeasy MinElute) [112], and we identified an *Iridoviridae* MAG (NCLDV MAG-2) that was detected in a single sample (S7). Ultimately, reconsidering these shared viral sequences alongside reagent negative controls is necessary to clarify their origin.

## Conclusions

Research on bryophyte viromes are scarce. In our samples the *Sphagnum* virome was compositionally heterogenous and only a small proportion of the total number of identified viral contigs were shared among all plants. While our findings suggest a diverse group of viruses and virus-like elements may comprise the *Sphagnum* core virome, further studies with more robust sampling and sequencing negative controls are needed to verify the presence and composition of a core virome as well as to address the effects of environmental factors on virome composition.

## Electronic supplementary material

Below is the link to the electronic supplementary material.

Supplementary Material 1

Supplementary Material 2

## Data Availability

All raw reads are available through NCBI under BioProject accession PRJNA1212510. The partitioned reads and assemblies analyzed in this study are available through Zenodo (https://zenodo.org/records/15281951).

## Abbreviations

MIUViG: Minimum Information about an Uncultivated Virus Genome
dsDNA: Double-stranded DNA
dsRNA: Double-stranded RNA
+ssRNA: Positive-sense single-stranded RNA
NCLDV: Nucleo-cytoplasmic large DNA virus
NCVOG: Nucleo-cytoplasmic virus orthologous groups
COG: Clusters of orthologous genes
RdRp: RNA-dependent RNA polymerase
MAG: Metagenome-assembled genome
