# Supplementary material for: Identification of shared viral sequences in peat moss metagenomes reveals elements of a possible Sphagnum core virome
Source: Environ Microbiome. 2025 Jun 5;20:62. doi: 10.1186/s40793-025-00719-0 (PMC12142844; doi:10.1186/s40793-025-00719-0)
Supplement: Supplementary file 1 — Supplementary Material 1 [file 40793_2025_719_MOESM1_ESM.pdf]

ADDITIONAL FILE 1

Identification of shared viral sequences in peat moss metagenomes reveals elements of a possible *Sphagnum* core virome

Elizabeth R. Denison[1], Helena L. Pound[1], Eric R. Gann[1], Naomi E. Gilbert[2], David J. Weston[3], Dale A. Pelletier[3] and Steven W. Wilhelm[1#]

1) Department of Microbiology, University of Tennessee, Knoxville, Tennessee, USA
2) Physical and Life Sciences Directorate, Lawrence Livermore National Laboratory, Livermore, CA, USA
3) Biosciences Division, Oak Ridge National Laboratory, Oak Ridge TN, USA

Supplemental Results

Details regarding RNA Virus Contig 6

RNA Virus Contig 6 contains a picobirnavirus-like RdRp, but the contig length (6.7 kb) and nine predicted ORFs is irregular for picobirnavirus genomes (Figure S10). *Picobirnaviridae* spp. have bisegmented genomes, where one segment encodes the RdRp plus one or two other ORFs (1). Unsegmented picobirnavirus-like sequences have also been described that encode the RdRp plus one or two additional ORFs (e.g., (2,3)).

Regarding RNA Virus Contig 6, no ORFs aside from the putative RdRp showed homology to known proteins in NCBI (i.e., no BLASTP hits against the NCBI non-redundant database (database accessed April 2025)). However, a NCBI Conserved Domain search showed that the ORF adjacent to the putative RdRp had a non-specific hit to a transposase domain (COG4584 superfamily, E-value: 1.66E-03, query cover: 25aa) (Figure S10). Additionally, a short region of the contig (129 nt) containing no predicted ORFs showed significant nucleotide similarity to *Sphagnum* genomes (top BLASTN hit to *S. jensenii* genome assembly, chromosome: 6; Accession: OZ020101.1; 96.15% identity; E-value: 2E-48). The contig aligned to a region of a feature (1.47% of the CDS) containing an unnamed protein product with a SIT4 phosphatase-associated protein domain (protein accession: CAK9274761.1; pfam04499).

We assessed if any metagenome reads mapped to RNA Virus Contig 6 (as it could indicate if it is a transcribed endogenized viral element in the *Sphagnum* or other organismal genome in our samples), but zero metagenome reads aligned to the contig (90% identity, 90% read length thresholds). Lastly, we confirmed that the virus-like region of the contig was present in all samples by mapping metatranscriptome reads to the RdRp CDS sequence only (i.e., to

check that the contig was not passing the 75% coverage threshold only due to reads mapping to the non-viral part of the contig) (data not shown).

Reference

1. Delmas, B., Attoui, H., Ghosh, S., Malik, Y.S., Mundt, E., Vakharia, V.N., et al. (2019). ICTV virus taxonomy profile: *Picobirnaviridae*. Journal of General Virology 100(2), 133-134. doi: https://doi.org/10.1099/jgv.0.001186.
2. Luo, X.L., Lu, S., Jin, D., Yang, J., Wu, S.S., and Xu, J. (2018). *Marmota himalayana* in the Qinghai-Tibetan plateau as a special host for bi-segmented and unsegmented picobirnaviruses. Emerg Microbes Infect 7(1), 20. doi: 10.1038/s41426-018-0020-6.
3. Shi, M., Lin, X.D., Tian, J.H., Chen, L.J., Chen, X., Li, C.X., et al. (2016). Redefining the invertebrate RNA virosphere. Nature 540(7634), 539-+. doi: 10.1038/nature20167.

Supplemental Figures

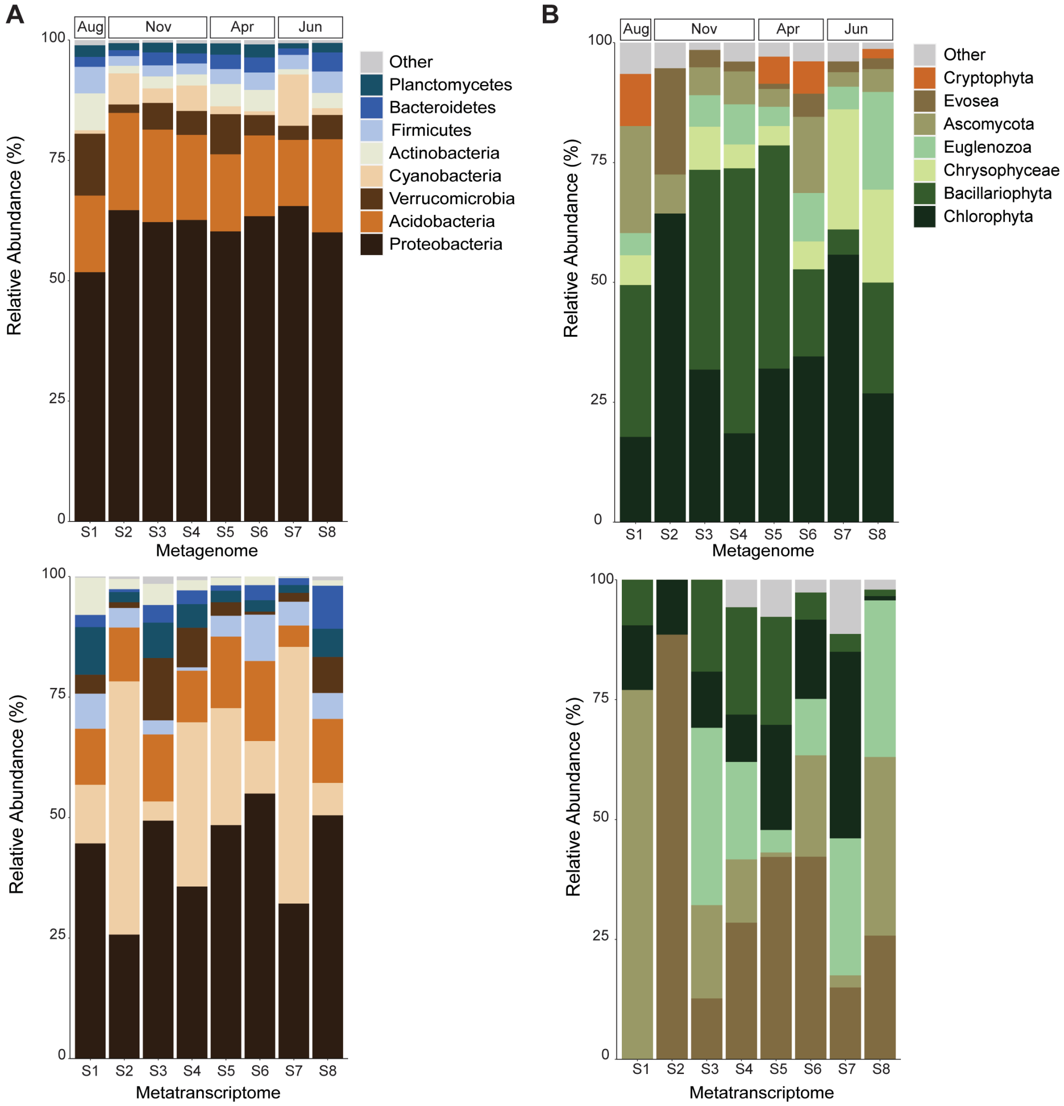

Figure S1. Relative abundance of microbial taxa in the metagenomes and metatranscriptomes. A) Estimate of prokaryotic abundance using mapped reads to RpoB-encoding contigs. B) Estimate of micro-eukaryotic abundance using RPB1. Abundance values were an average of the two replicates for metatranscriptomes. Minor prokaryotic taxa include: *Armatimonadetes*, *Chlamydiae*, *Gemmatimonadetes*, *Tenericutes*, *Euryarchaeota*, and unclassified bacteria. Minor abundance eukaryotic taxa include: Basidiomycota, Ciliophora, and Ichthyosporea.

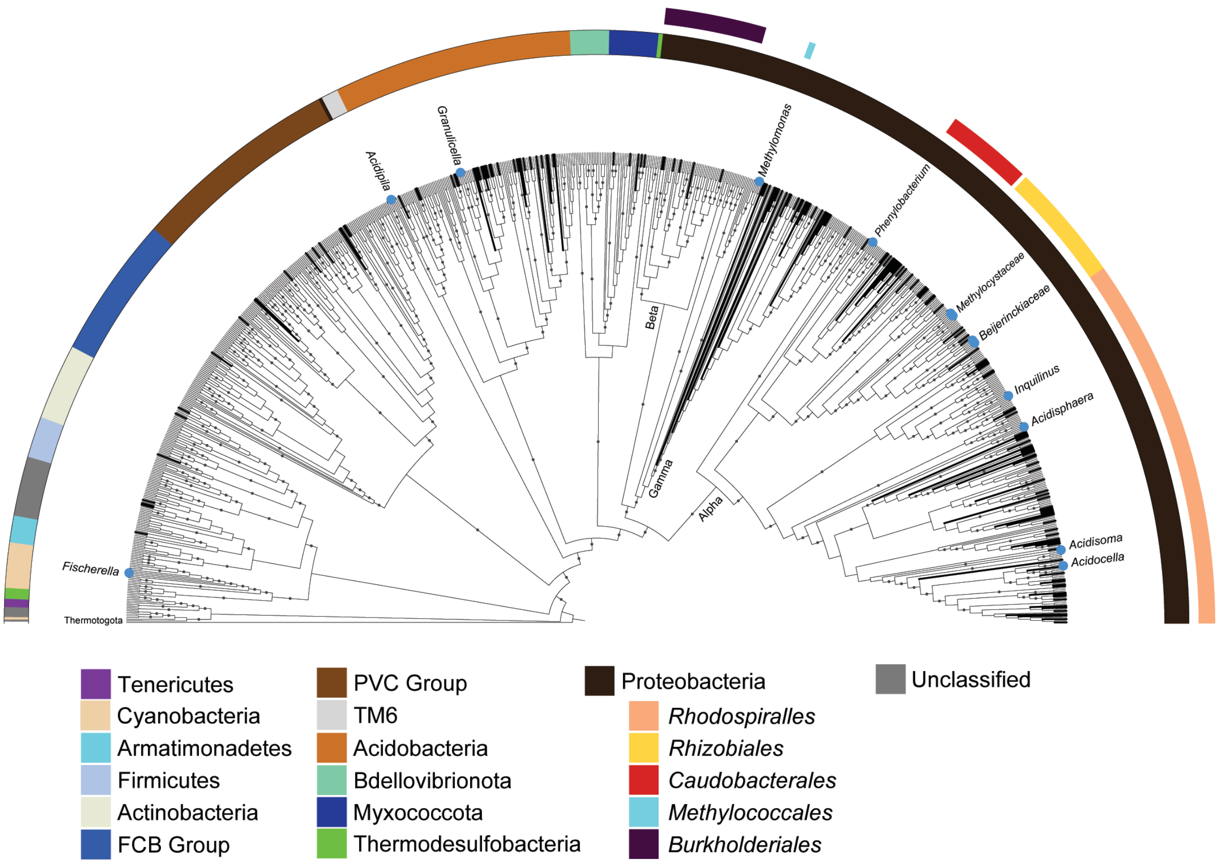

Figure S2. Cladogram of maximum likelihood phylogeny of predicted RpoB protein sequences. Core taxa identified in Kolton *et al*. (2022) are labeled and indicated by nodes ending in circles. Core RpoB detected in this dataset are bolded branches Inner ring represents prokaryotic phylum. Order is shown for clades of interest containing core prokaryotic members. Abundance for each RpoB is represented as average coverage. FastTree bootstraps > 0.9 are displayed as dots.

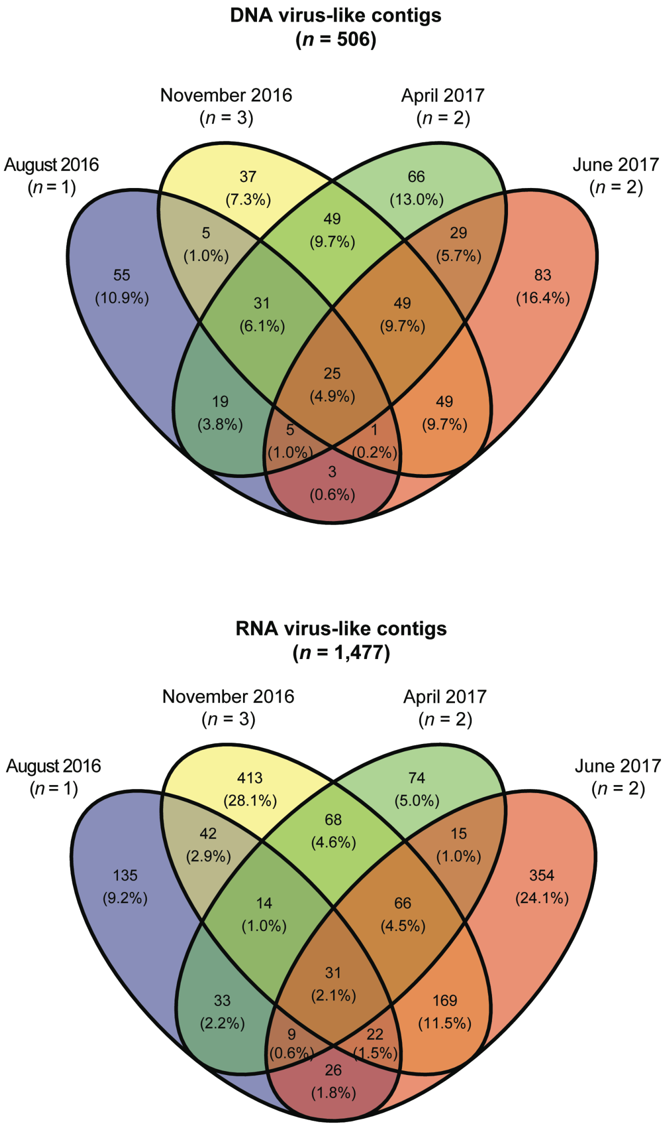

Figure S3. Venn diagrams showing the number of shared and unique virus-like contigs between plants grouped by sampling month. The number of samples per month is shown in parentheses.

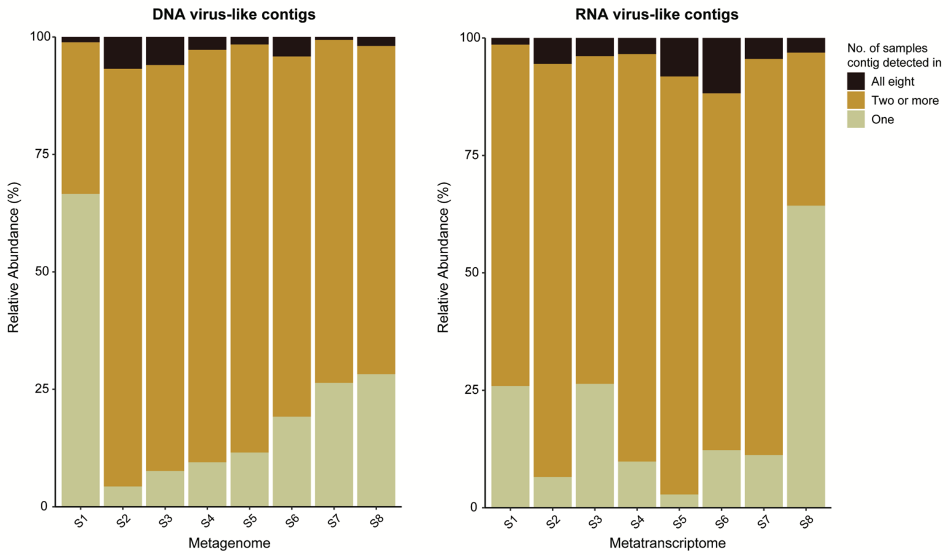

Figure S4. Relative abundance of the virus-like contigs grouped by sharing pattern (contigs present in all eight, two or more, or only one sample(s)). Relative abundance values here were based on the number of reads mapped to contigs and normalized by contig length. For metatranscriptomes, the read count values were averaged between two replicates.

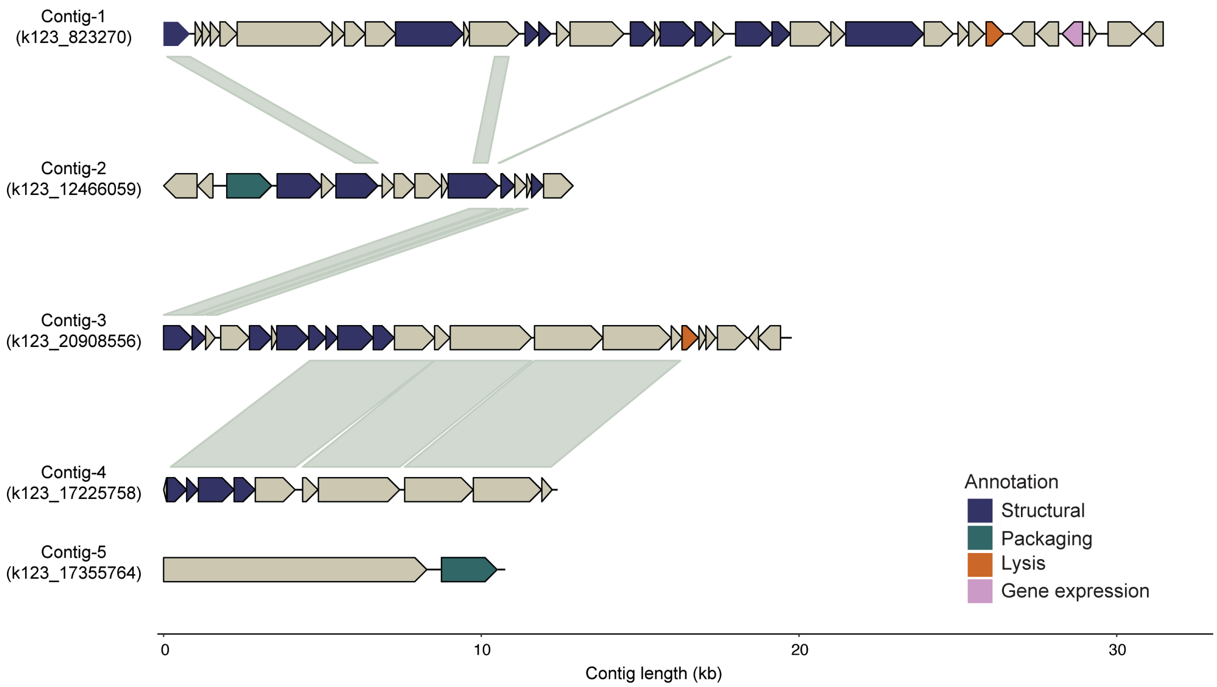

Figure S5. Visualization of nucleotide similarity between the shared DNA virus contigs. The BLASTN table used to construct his figure is available in Table S8.

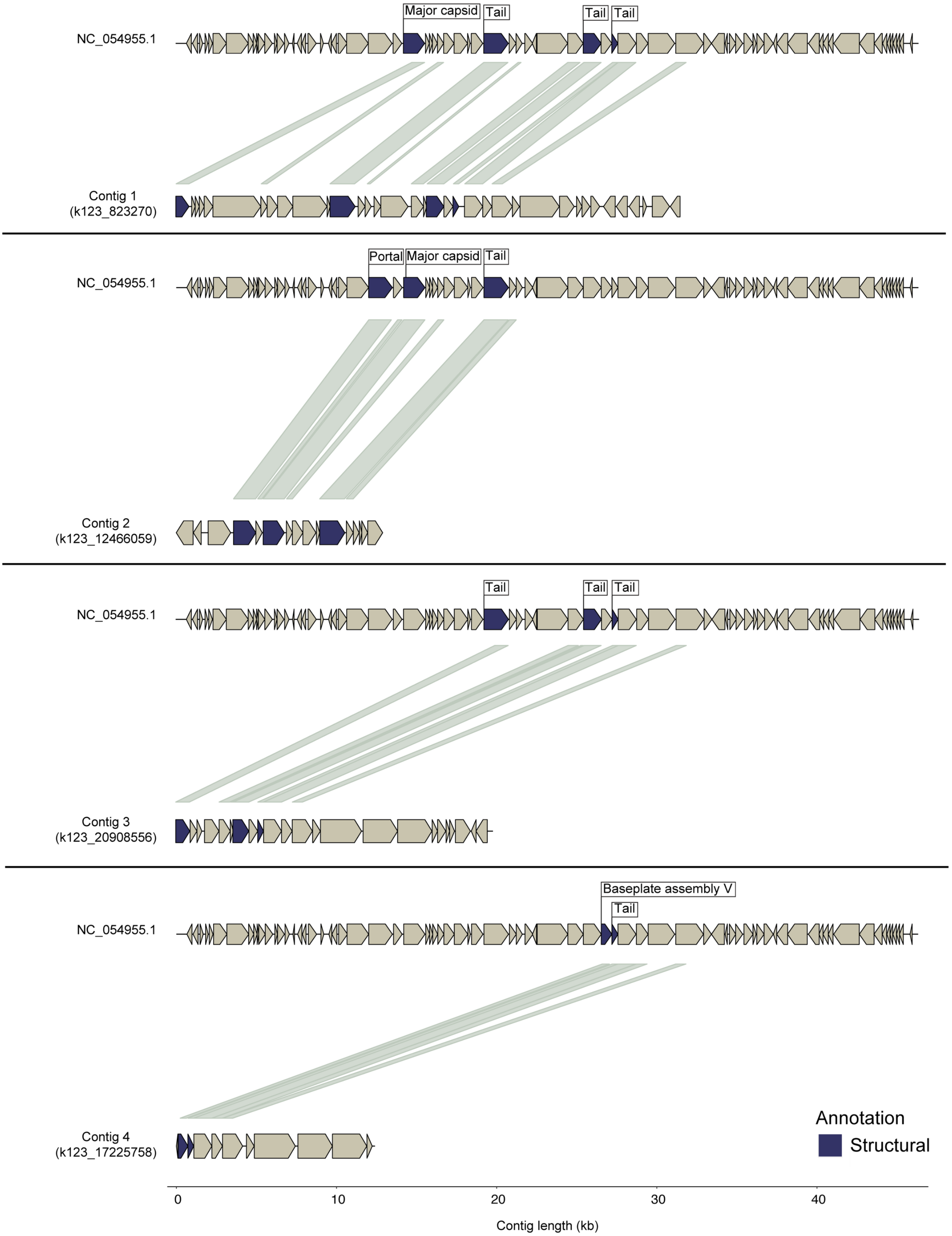

Figure S6. Visualization of protein sequence similarity between the shared DNA virus contigs and Ralstonia phage Raharianne (NC_054955.1). The BLASTP table used to construct this figure is available in Table S9.

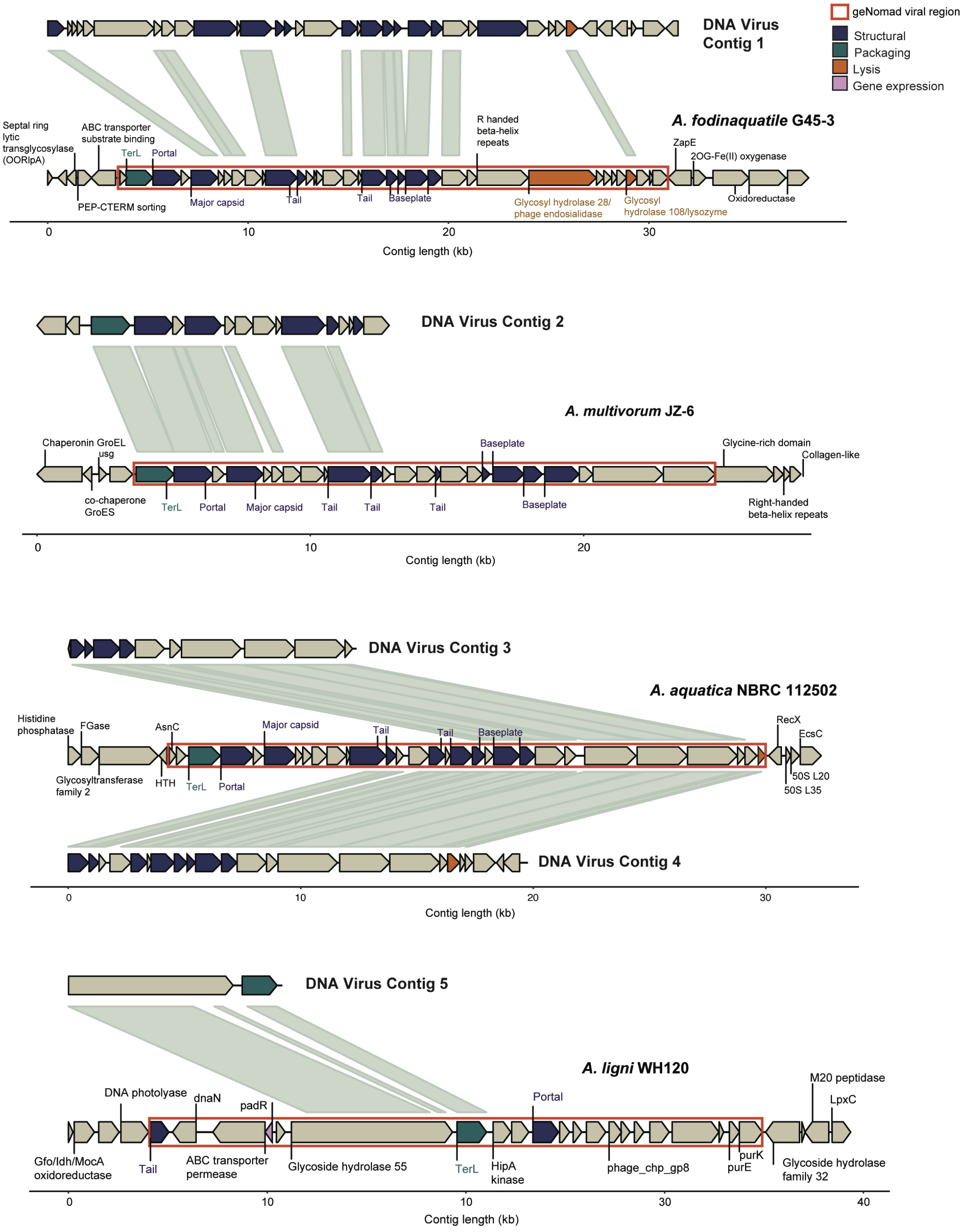

Figure S7. Visualization of protein sequence similarity and annotations between the shared DNA virus contigs and the top hit RefSeq bacterial genome.

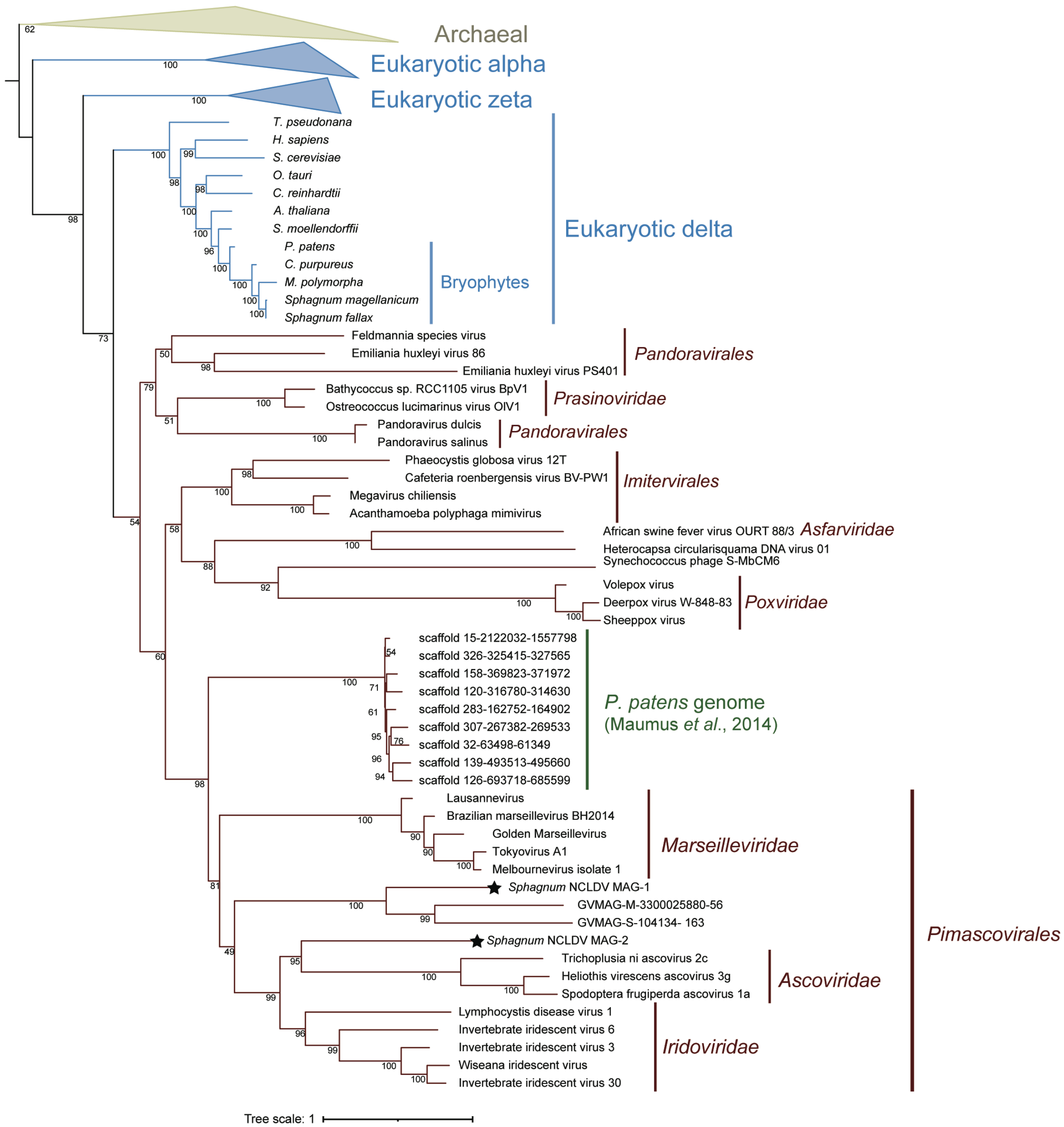

Figure S8. Maximum likelihood phylogeny of DNA polymerase protein sequences. The tree was inferred using the best-fit modeldetermined by IQTREE (Q.pfam+F+I+G4). DNA polymerase sequences from the *Sphagnum* NCLDV MAGs, *S. fallax* and *S. magellanicum* genomes, and additional *Pimascovirales* genomes were aligned to the alignment retrieved from Maumus *et al.* (2014).

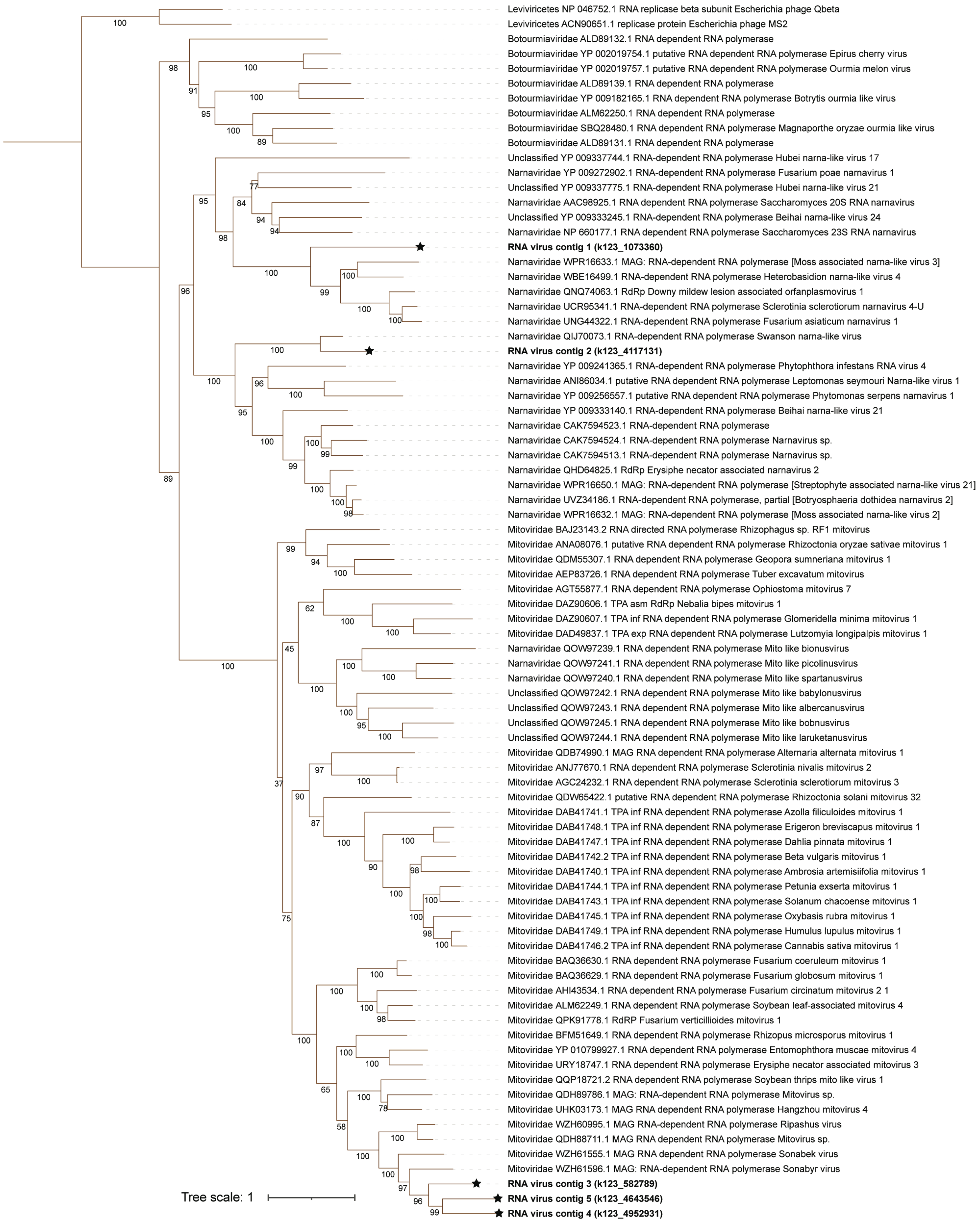

Figure S9. Full version of the RdRp trees presented in Figure 5 with the collapsed clades expanded. (A) *Lenarviricota* (B) *Durnavirales* (C) *Picornavirales* (D) *Hepelivirales*

B

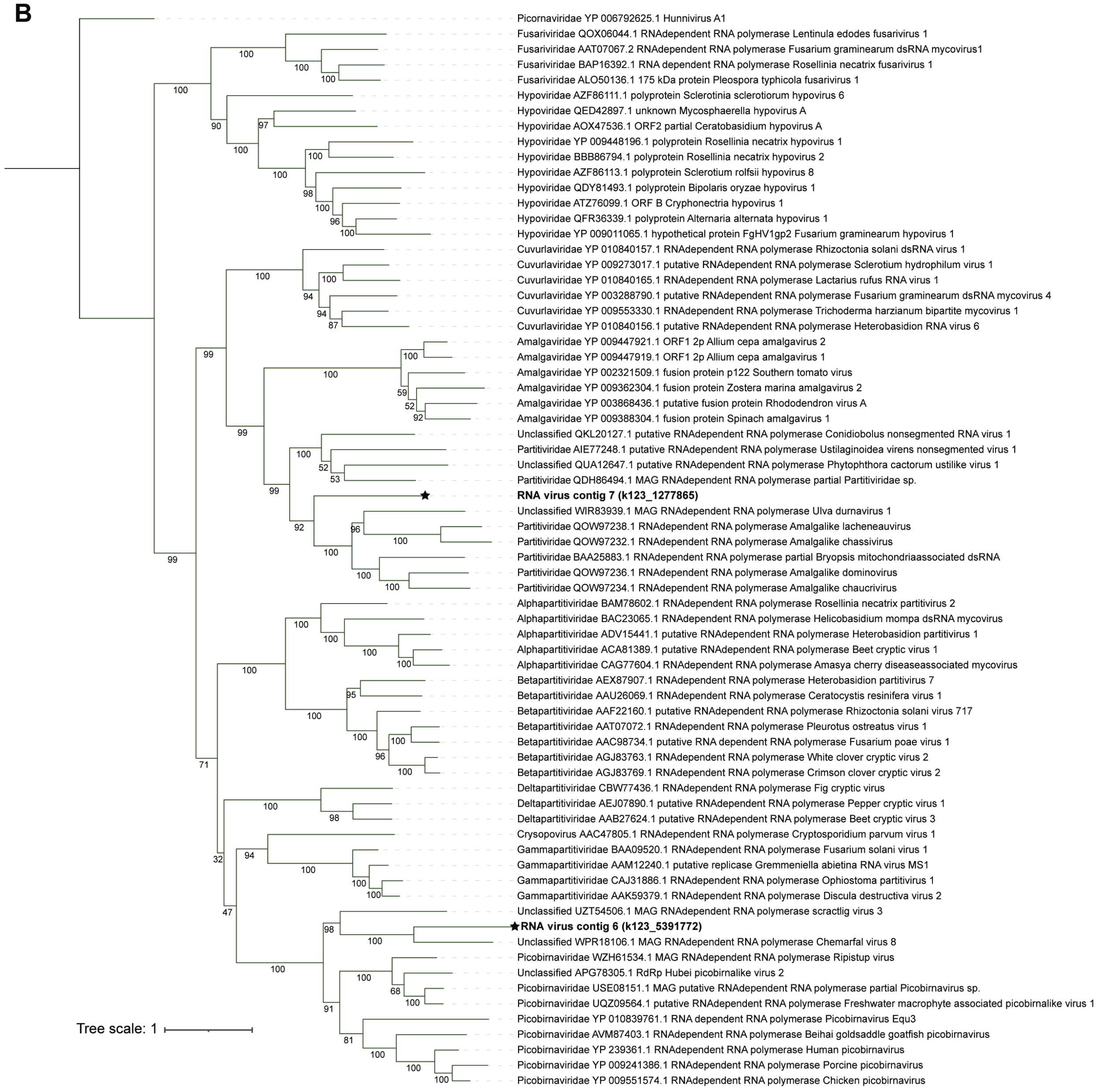

Figure S9 continued. Full version of the RdRp trees presented in Figure 5 with the collapsed clades expanded. (A) *Lenarviricota* (B) *Durnavirales* (C) *Picornavirales* (D) *Hepelivirales*

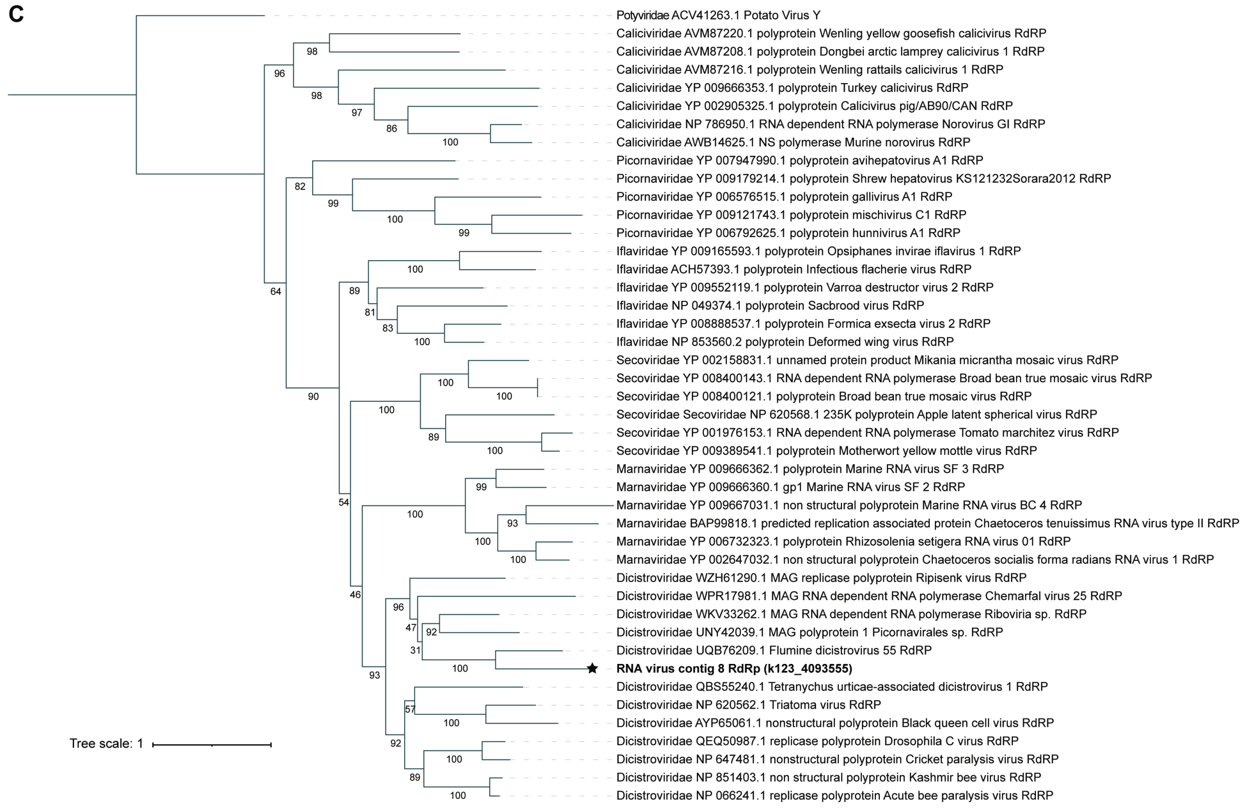

Figure S9 continued. Full version of the RdRp trees presented in Figure 5 with the collapsed clades expanded. (A) *Lenarviricota* (B) *Durnavirales* (C) *Picornavirales* (D) *Hepelivirales*

D

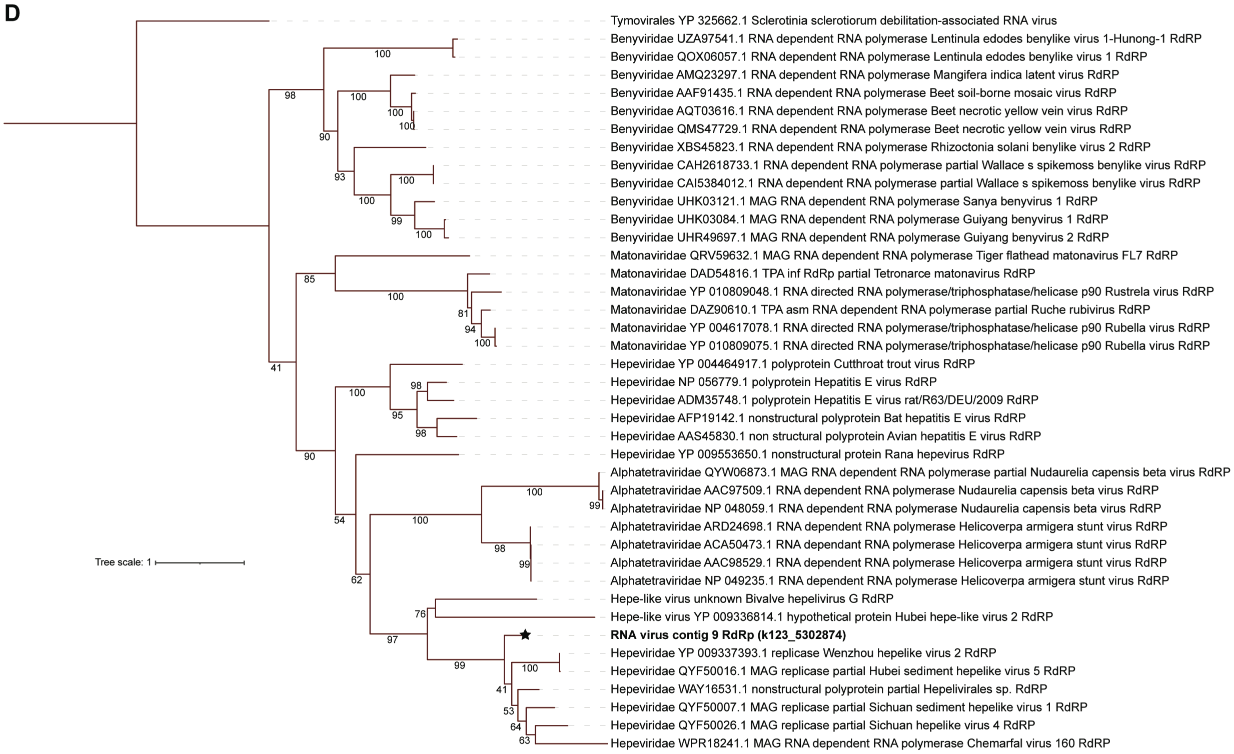

Figure S9 continued. Full version of the RdRp trees presented in Figure 5 with the collapsed clades expanded. (A) *Lenarviricota* (B) *Durnavirales* (C) *Picornavirales* (D) *Hepelivirales*

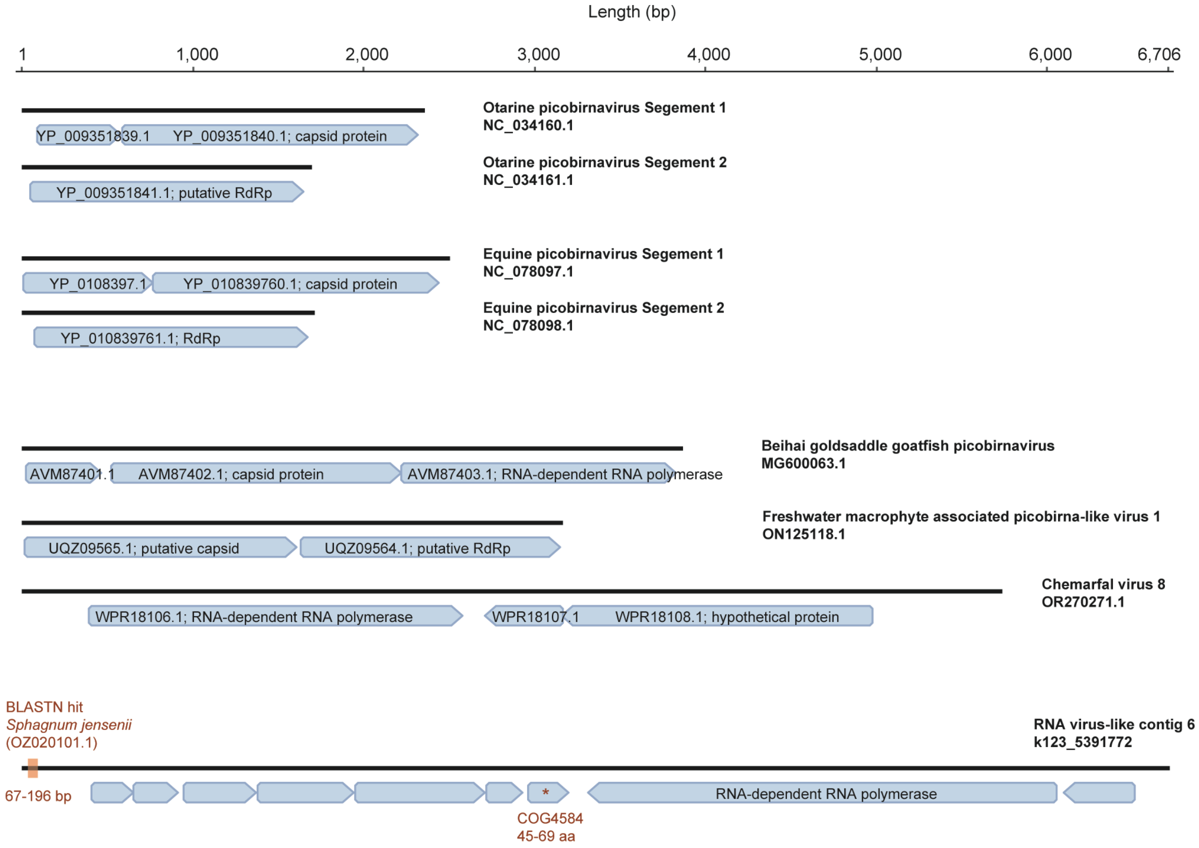

Figure S10. A) Examples of *Picobirnaviridae* and picobirnavirus-like genome organization relative to RNA virus-like contig 6. Annotations were retrieved from NCBI. The region of similarity between RNA virus-like contig 6 and the *Sphagnum jensenii* chromosome is highlighted in orange (exact coordinates are listed in base pairs (bp), orange box is not to scale). The ORF containing a transposase (COG4584) non-specific domain hit is noted by an asterisk and the hit coordinates provided in amino acids (aa).
